# The Nek2 centrosome-mitotic kinase contributes to the mesenchymal state, cell invasion, and migration of triple-negative breast cancer cells

**DOI:** 10.1038/s41598-021-88512-0

**Published:** 2021-04-27

**Authors:** Yainyrette Rivera-Rivera, Mihaela Marina, Shirley Jusino, Miyoung Lee, Jaleisha Vélez Velázquez, Camille Chardón-Colón, Geraldine Vargas, Jaya Padmanabhan, Srikumar P. Chellappan, Harold I. Saavedra

**Affiliations:** 1 Division of Pharmacology and Cancer Biology, Department of Basic Sciences, Ponce Health Sciences University/Ponce Research Institute, PO Box 7004, Ponce, 00716-2348 Puerto Rico; 2 MediTech Media, Two Ravinia Drive, Suite 605, Atlanta, GA 30346 USA; 3 Department of Pediatrics, Aflac Cancer and Blood Disorder Center, Emory University School of Medicine, Atlanta, GA 30322 USA; 4 Department of Biology, University of Puerto Rico-Ponce, 2151 Santiago de los Caballeros Avenue, Ponce, 00716 Puerto Rico; 5 Department of Tumor Biology, H. Lee Moffitt Cancer Center and Research Institute, 12902 USF Magnolia Drive, Tampa, FL 33612 USA

**Keywords:** Cancer, Cell biology

## Abstract

Nek2 (NIMA‐related kinase 2) is a serine/threonine-protein kinase that localizes to centrosomes and kinetochores, controlling centrosome separation, chromosome attachments to kinetochores, and the spindle assembly checkpoint. These processes prevent centrosome amplification (CA), mitotic dysfunction, and chromosome instability (CIN). Our group and others have suggested that Nek2 maintains high levels of CA/CIN, tumor growth, and drug resistance. We identified that Nek2 overexpression correlates with poor survival of breast cancer. However, the mechanisms driving these phenotypes are unknown. We now report that overexpression of Nek2 in MCF10A cells drives CA/CIN and aneuploidy. Besides, enhanced levels of Nek2 results in larger 3D acinar structures, but could not initiate tumors in a p53^+/+^ or a p53^−/−^ xenograft model. Nek2 overexpression induced the epithelial-to-mesenchymal transition (EMT) while its downregulation reduced the expression of the mesenchymal marker vimentin. Furthermore, either siRNA-mediated downregulation or INH6’s chemical inhibition of Nek2 in MDA-MB-231 and Hs578t cells showed important EMT changes and decreased invasion and migration. We also showed that Slug and Zeb1 are involved in Nek2 mediated EMT, invasion, and migration. Besides its role in CA/CIN, Nek2 contributes to breast cancer progression through a novel EMT mediated mechanism.

## Introduction

Since chromosome instability (CIN, or the active generation of structural and numerical chromosome changes)^1,2^ accelerates tumor evolution, cells have developed mechanisms to suppress CIN, including the tight regulation of centrosome duplication and mitosis^1,3–13^. When these processes are de-regulated^14–21^, centrosome amplification (CA, or the acquisition of ≥ 3 centrosomes per cell) arises and drives mitotic perturbations, aneuploidy, defective polarity, and CIN^14,22–28^. Present in pre-malignant mammary lesions and tumors, CA is more prevalent in triple-negative (estrogen and progesterone receptor-negative and non-amplified for Her2, or ER-PR-Her2-) breast cancer subtype and correlates with stage, grade, and poor survival of breast cancer patients^29–36^. While low levels of CA and CIN promote spontaneous tumor formation in mice (not including mammary tumors), high levels are intolerable, resulting in cell cycle arrest or cell death^12,13,37,38^.

Nek2 is a mitotic serine/threonine-protein kinase that localizes to centrosomes and kinetochores, where it controls centrosome separation, bipolar spindle formation, kinetochore-spindle attachments, and the spindle assembly checkpoint (SAC)^39–41^. High levels of Nek2 mRNA and protein were first detected in breast tumors^24^ and its overexpression correlates with poor prognosis of breast cancer patients^42–44^. Nek2 inhibition decreases tumor cell proliferation and resensitizes cancer cells to chemotherapeutic agents^26,45–49^. Nek2 may participate in transformation and tumor progression by modulating other cancer-promoting signaling pathways, such as Akt, Rho, E-cadherin, β-catenin, or MAPK^50–55^.

Nek2 is one of the proteins associated with lung metastasis in a breast cancer mouse model^42^ and is one of the top deregulated genes in metastatic lung cancer^48^. We demonstrated that Nek2 induces CA and invasive protrusions in Her2^+^ organoids lacking E2F3^56^, suggesting that unregulated Nek2 can influence early intermediates to metastasis, perhaps by inducing the epithelial-to-mesenchymal transition (EMT). EMT is initiated by the loss of epithelial junctions and cell polarity, which results in cytoskeleton reorganization and a mesenchymal phenotype and occurs in gradual (hybrid) steps^57–62^. EMT involves the loss of the epithelial marker E-cadherin, increases in mesenchymal markers (e.g. vimentin), and matrix metalloproteases (MMPs), which facilitates cell migration, invasion, and extracellular matrix degradation^63–67^. EMT is mediated by transcription factors that belong to the SNAIL (SNAI 1–3), bHLH (Twist1 and Twist2), and the ZEB (Zeb1-2) families^60^. Studies performed in several cancer models have shown that Nek2 overexpression correlates with EMT markers^68^. However, none of these studies included breast cancer; therefore, here we explore the role of Nek2 in driving breast cancer EMT.

## Results

### Nek2 is overexpressed in multiple breast cancer subtypes and correlates with poor survival

To identify the molecular subtypes of breast cancer that overexpress Nek2, we performed bioinformatic analyses using the METABRIC database of 1904 patients with breast cancer^69,70^. Because Luminal A has the best prognosis of all breast cancer subtypes^71^, we compared the log intensity of Nek2 mRNA expression in several breast cancer subtypes relative to Luminal A. This analysis indicates that Nek2 levels are significantly higher in Basal, Her2^+^, Luminal B, and Claudin-Low relative to Luminal A (Supp. Fig. 1). When expressed using percentages of patients, 10.1% of basal, 5.2% of Luminal B, 4.5% of Her2^+^, 2.5% of Claudin-Low, and none of the Luminal A and Normal-like breast cancers overexpressed Nek2 (Supp. Table 1). METABRIC also showed frequent gene amplification of Nek2 in all subtypes.

**Figure 1:**
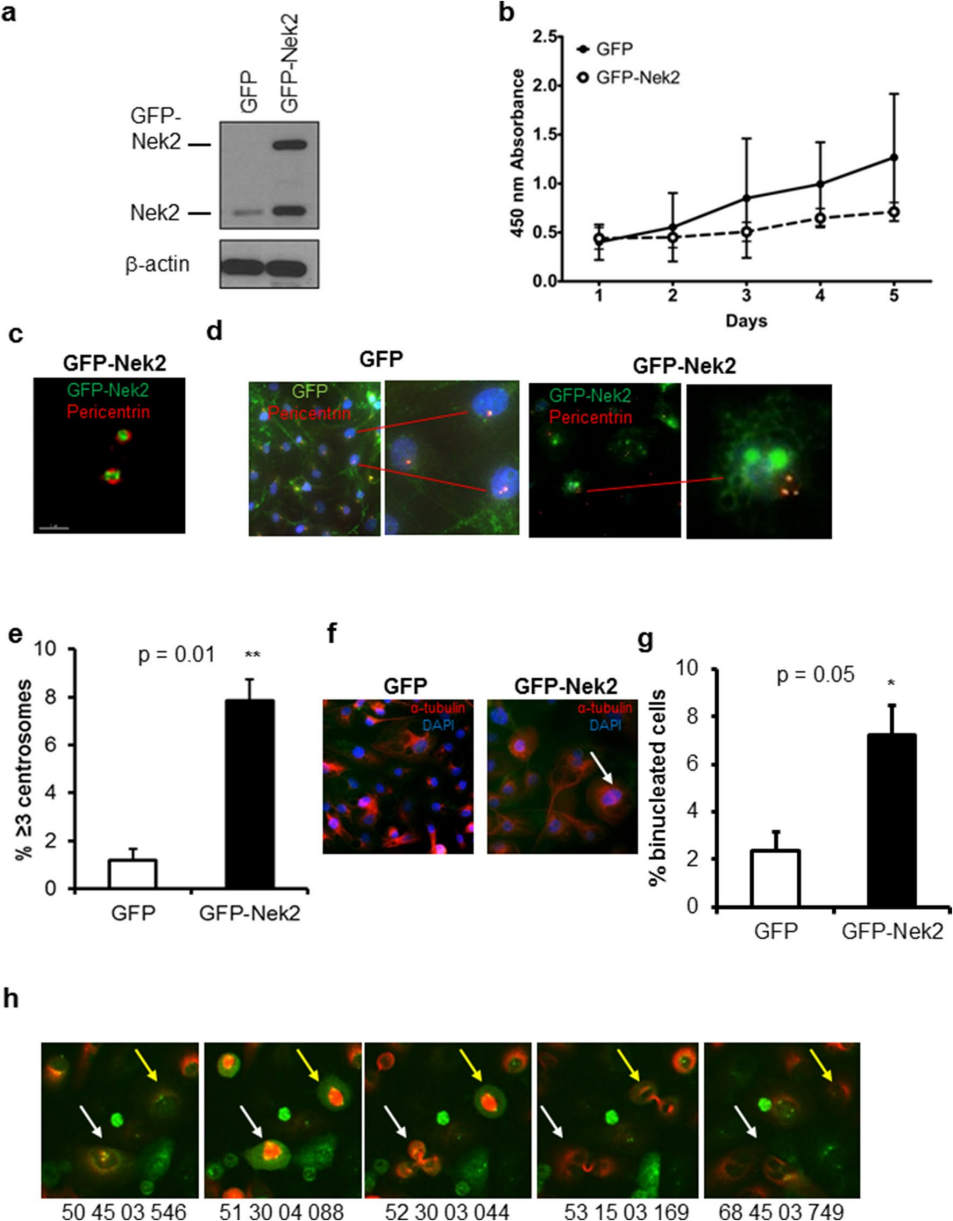
Nek2 overexpression causes centrosome amplification and binucleation. (**a**) Representative immunoblot of MCF10A cells expressing either GFP or GFP-Nek2 indicating overexpression of endogenous Nek2 and GFP-Nek2 detected with Nek2 antibodies. (**b**) CCK8 **c**ell growth/viability assay comparing MCF10A expressing GFP or GFP-Nek2. (**c**) High-resolution microscopy of a centrosome pair indicating localization of pericentrin (red) and GFP-Nek2 (green). (**d**) Immunofluorescent staining of pericentrin (red) in GFP and GFP-Nek2-expressing cells (green) counterstained with DAPI (blue). The right inset (GFP-Nek2) indicates a cell with 3 centrosomes. (**e**) Quantifications of fluorescent microscopy for centrosome amplification by pericentrin staining. N = 3, bars = mean ± SD, *p < 0.05. (**f**) Immunofluorescent staining of α-tubulin (red) in GFP and GFP-Nek2-expressing cells counterstained with DAPI (blue). The arrow indicates a binucleated cell. (**g**) Quantifications of fluorescent microscopy for binucleation by α-tubulin staining. N = 3, bars = mean ± SD, *p < 0.05. (**h**) Representative screenshots of MCF10A/GFP-Nek2 cells subjected to live imaging for 72 h. Centrosome-localized GFP-Nek2 is visible in green and the microtubules are marked by RFP-α-tubulin. Arrows indicate tripolar cell division at different time points.

### Nek2 overexpression causes centrosome amplification and chromosome instability

Because Nek2 is altered in breast cancers by overexpression and/or gene amplification, we tested if the overexpression of GFP-Nek2 is sufficient to trigger CA and binucleation in MCF10A, an established, p53-wild-type, a non-transformed mammary epithelial cell line with low percentages of CA and CIN^22,23,36,43,72^. GFP was used as a control (Fig. 1a). A cell growth/viability assay did not show significant differences in growth rates between GFP and GFP-Nek2 cells (Fig. 1b). However, the trend that GFP-Nek2 cells grow slower may be explained by the induction of CA/CIN that perhaps is leading to 5% cell death in the population (Supp. Table 2). High-resolution microscopy showed co-localization of GFP-tagged Nek2 with the centrosome marker pericentrin, indicating that GFP-Nek2 localizes to its native organelle (Fig. 1c). CA and binucleation assessed by pericentrin and α-tubulin immunofluorescence, respectively (Fig. 1d,f), were significantly higher in GFP-Nek2 cells (8% of cells for both) compared with controls (Fig. 1e,g). To examine single-cell fate, cells expressing GFP or GFP-Nek2 were transduced with RFP-α-tubulin lentiviral particles to observe mitotic spindles and followed for two cell cycles using live imaging (Fig. 1h). We observed the de novo formation of multipolar mitosis from a cell with pre-existing CA, and a cell with CA that resulted in normal mitosis; other observations included cells that underwent mitotic catastrophe (Supp. Table 2). Consistent with similar studies that overexpressed other mitotic kinases such as Aurora kinase A^73^, the overexpression of Nek2 triggered cytokinesis defects that resulted in binucleation.

To investigate if CA results in CIN, we used the micronucleus assay, which measures whole or partial missegregated chromosomes^74^. Micronuclei can greatly accelerate tumor evolution by replicating and recombining with genomic DNA^75^. The baseline level of micronucleation was 1% with controls vs 10% with GFP-Nek2 (Supp. Fig. 2a,b). Nek2 overexpression also resulted in chromosome losses and gains, with 68% of cells displaying a range of 51–60 chromosomes per cell compared with over 80% displaying 41–50 chromosomes per cell in GFP controls (Supp. Fig. 2c,d). We conclude that Nek2 overexpression is sufficient to trigger aneuploidy and CIN in non-tumorigenic cells.

**Figure 2:**
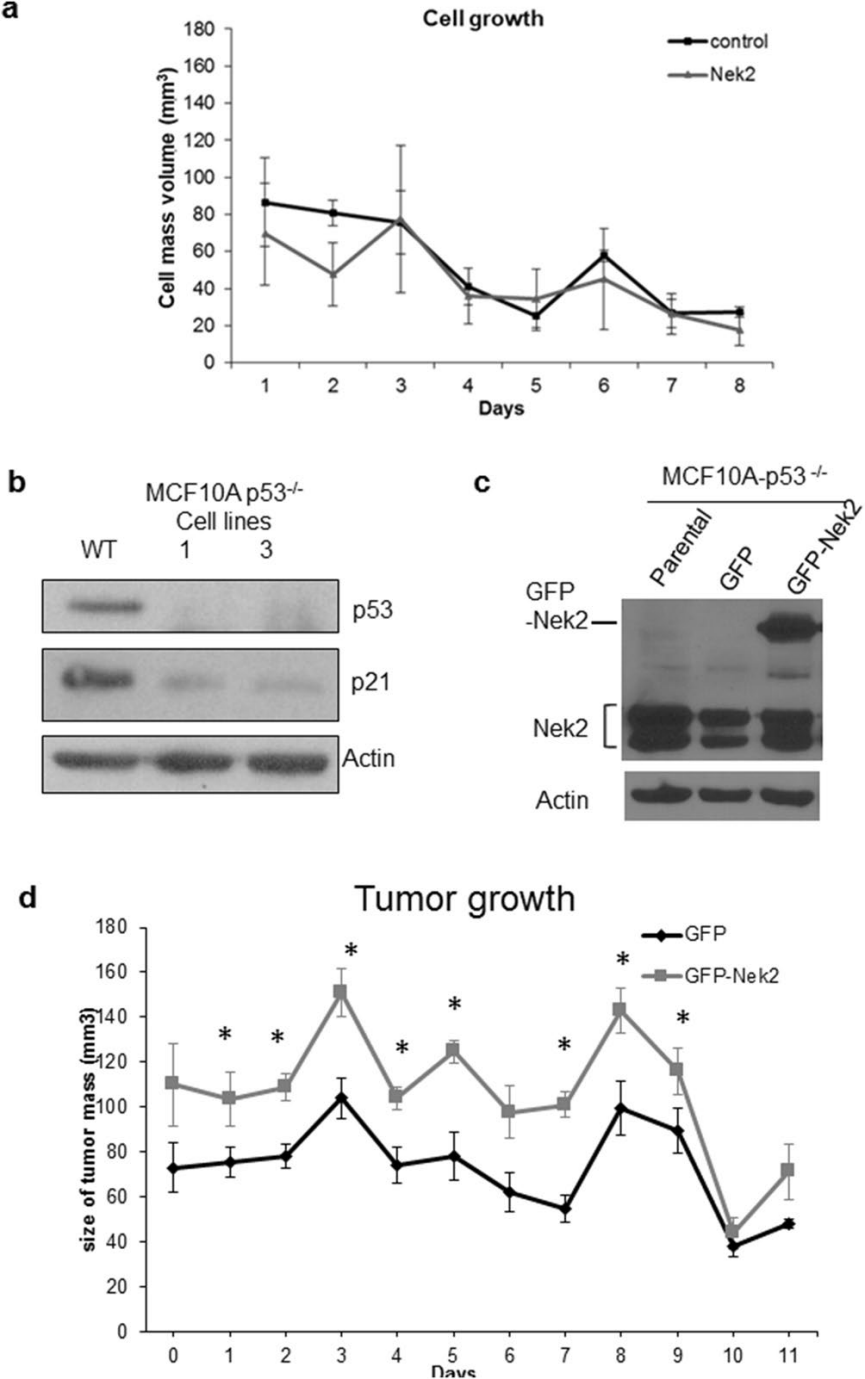
Nek2 overexpression does not promote tumorigenesis. (**a**) Growth curve of MCF10A control or overexpressing Nek2. (**b,c**) Immunoblots for p53 or p21Waf1 (**b**) or Nek2 protein levels (**c**) in MCF10A-p53^-/-^ parental, GFP, and GFP-Nek2 cells. (**d**) Tumor growth curve of MCF10A with GFP or GFP-Nek2. Bars = mean ± SD, *p < 0.05.

### Nek2 overexpression leads to larger acinar structures but is not sufficient to initiate tumorigenesis

To address the role of Nek2 in facilitating additional cancer-promoting phenotypes, MCF10A/GFP-Nek2 cells and controls were plated in matrigel 10 days until they formed acini. Visual examination revealed larger, acinar structures upon Nek2 overexpression (Supp. Fig. 3a). Quantification of overall volumes indicated significantly larger organoids in MCF10A/GFP-Nek2 3D cultures compared with control (Supp. Fig. 3b). This ability to trigger acinar growth in matrigel culture is indicative of the transforming properties that Nek2 might have.

**Figure 3:**
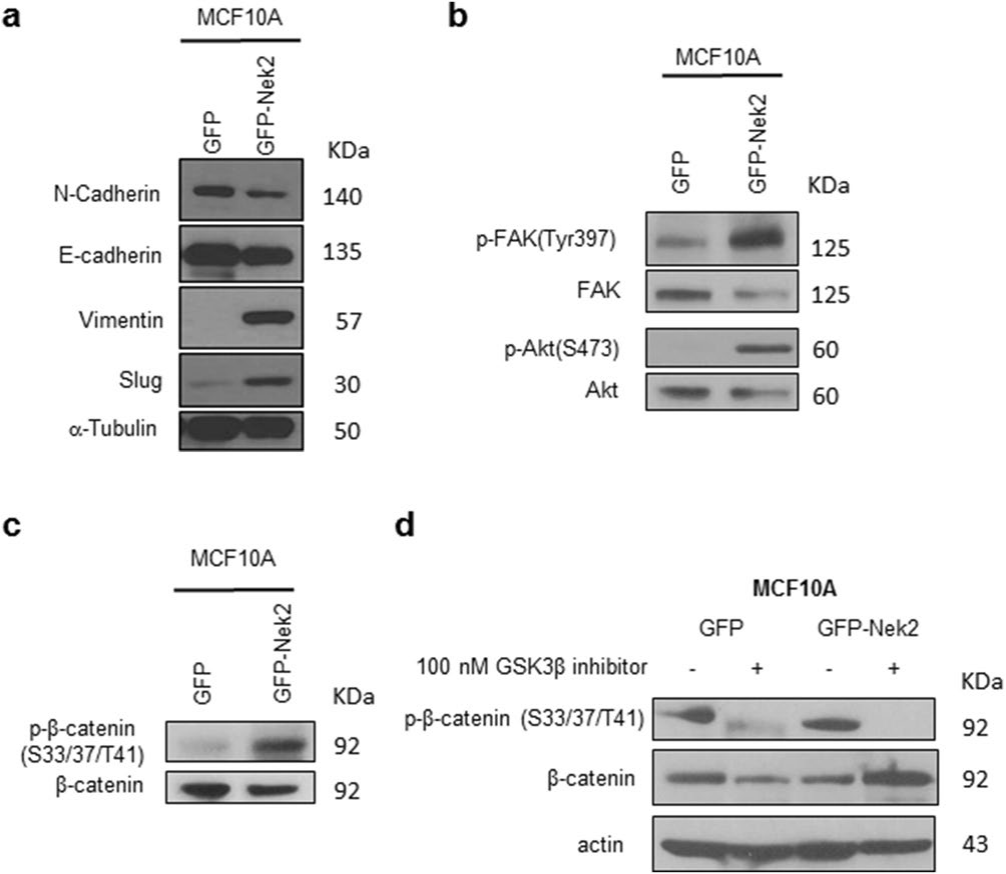
Nek2 regulates the epithelial-to-mesenchymal transition. (**a**) Immunoblotting of EMT pathway proteins. (**b**) Immunoblotting of phospho- and total Akt and FAK. (**c**) Immunoblotting of phospho- and total b-catenin in the presence or absence of inhibitor (**d**).

Previously, we showed that HCC1954 Her2^+^ breast cancer cells grow exponentially and form tumors 7 days after mammary fat pad injections into mice^76^. Using the same protocol, we found that GFP-Nek2 did not initiate tumors in MCF-10A cells (Fig. 2a). Since the p53 pathway is a suppressor of CIN and CA^77–79^, we addressed if GFP-Nek2 can induce tumorigenesis in MCF10A cells with a somatic knockout of p53^80^ (Fig. 2b,c). While the volume of the mammary glands in cells expressing GFP-Nek2 in p53-null cells was significantly larger than these expressing GFP, no stable tumors were observed (Fig. 2d).

### Nek2 triggers the epithelial-to-mesenchymal transition in mammary epithelial cells

Since Nek2 is not sufficient to initiate tumorigenesis, we asked if it can affect other malignant features of cancer cells. To test the effects of Nek2 overexpression on cell spreading, cells were plated and imaged 45 and 90 min after seeding. Strikingly, we detected that MCF10A/GFP-Nek2 cells formed lamellipodia and filopodia as early as 45 min post-seeding, whereas GFP control cells remained round with smooth membrane edges until 90 min post-plating (Supp. Fig. 4a). Quantification of cell spreading indicated that the GFP-Nek2 cells displayed significantly larger areas and perimeters compared with GFP control during cell attachment (Supp. Fig. 4b,c).

**Figure 4:**
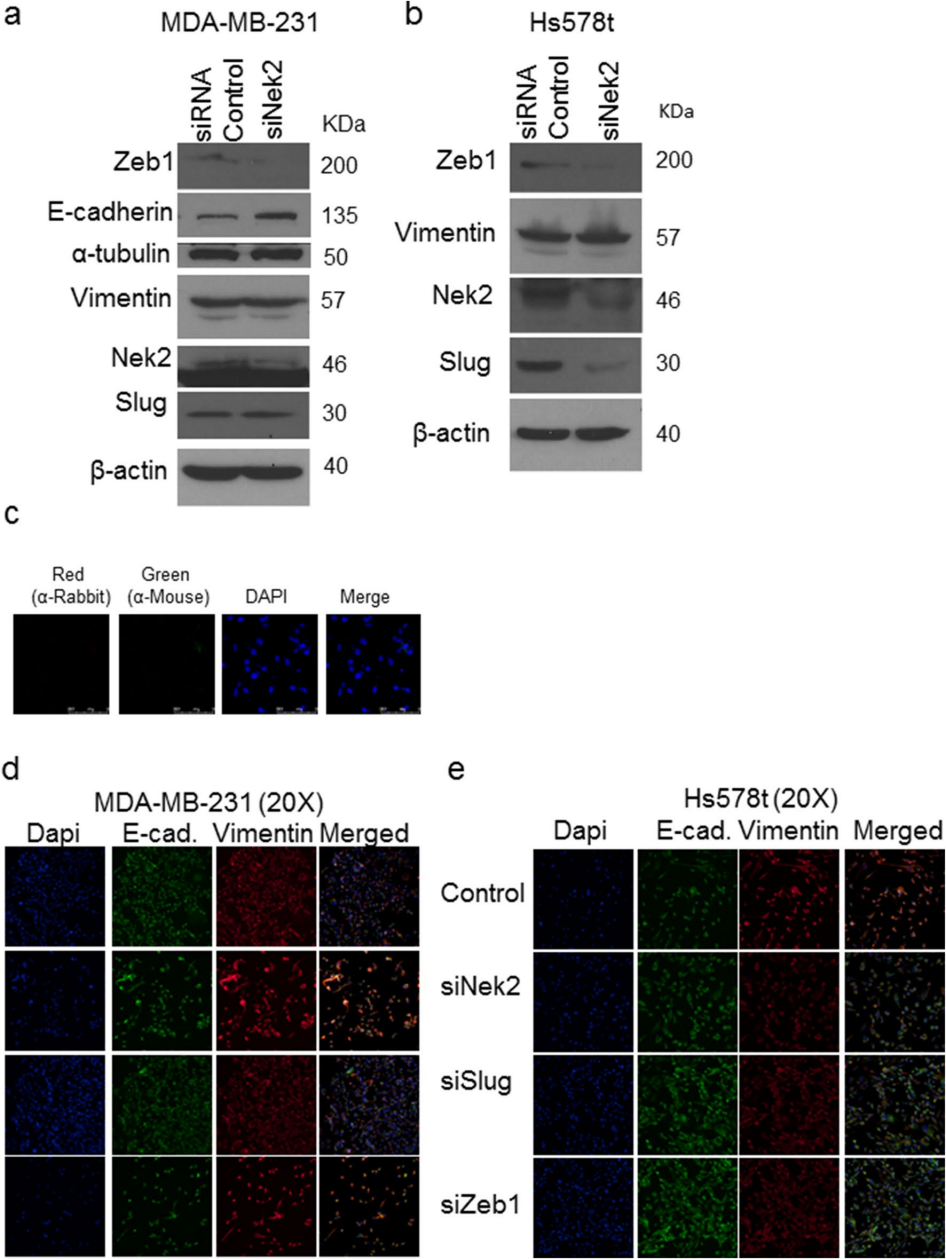
Nek2 maintains the mesenchymal state in triple-negative breast cancer cells. (**a,b**) Immunoblots of EMT pathway markers in MDA-MB-231 treated with 50 nM siNek2 5-siRNA pools (Dharmacon) in MDA-MB-231 cells (**a**) and Hs578t cells (**b**). (**c**) Immunofluorescence controls with secondary antibodies. (**d,e**) Co-Immunofluorescent staining of E-cadherin (green) and Vimentin in MDA-MB-231 cells (**d**) or Hs578t cells (**e**), in cells transfected with 50 nM of the indicated 5-siRNA pools (Dharmacon). Nucleus was counterstained with DAPI (blue).

To establish if there are predicted and/or already known correlations between Nek2 and important EMT markers in this pre-metastatic process, we performed in silico analysis using the online tool STRING^81^. We explored correlations between Nek2 and β-catenin (CTNNB1), E-cadherin (CDH1), N-cadherin (CDH2), vimentin (VIM), Zeb1 (ZEB1), Snail (SNAI1), Slug (SNAI2), and ZO-1 (TJP1). Of these, Nek2 has been directly linked to only CTNNB1 and CDH1 through phosphorylation and reduction of protein levels, respectively^52,55^. The molecular action of most remaining EMT proteins in the network is binding and/or transcriptional regulation (Supp. Fig. 5a). When we analyzed the strength of the interaction, we detected medium confidence (0.400) between Nek2 and CDH1 and high confidence (0.700) between Nek2 and CTNNB1 (Supp. Fig. 5b). Furthermore, the type of protein–protein interaction within the network showed that there are text mining and coexpression evidence between Nek2 and CDH1, while there is text mining as well as experimentally determined evidence between Nek2 and CTNNB1 (Supp. Fig. 3c). The types of interactions among the remaining EMT proteins in the network were mainly through text mining, co-expression, experimentally determined, and/or from curated databases.

**Figure 5:**
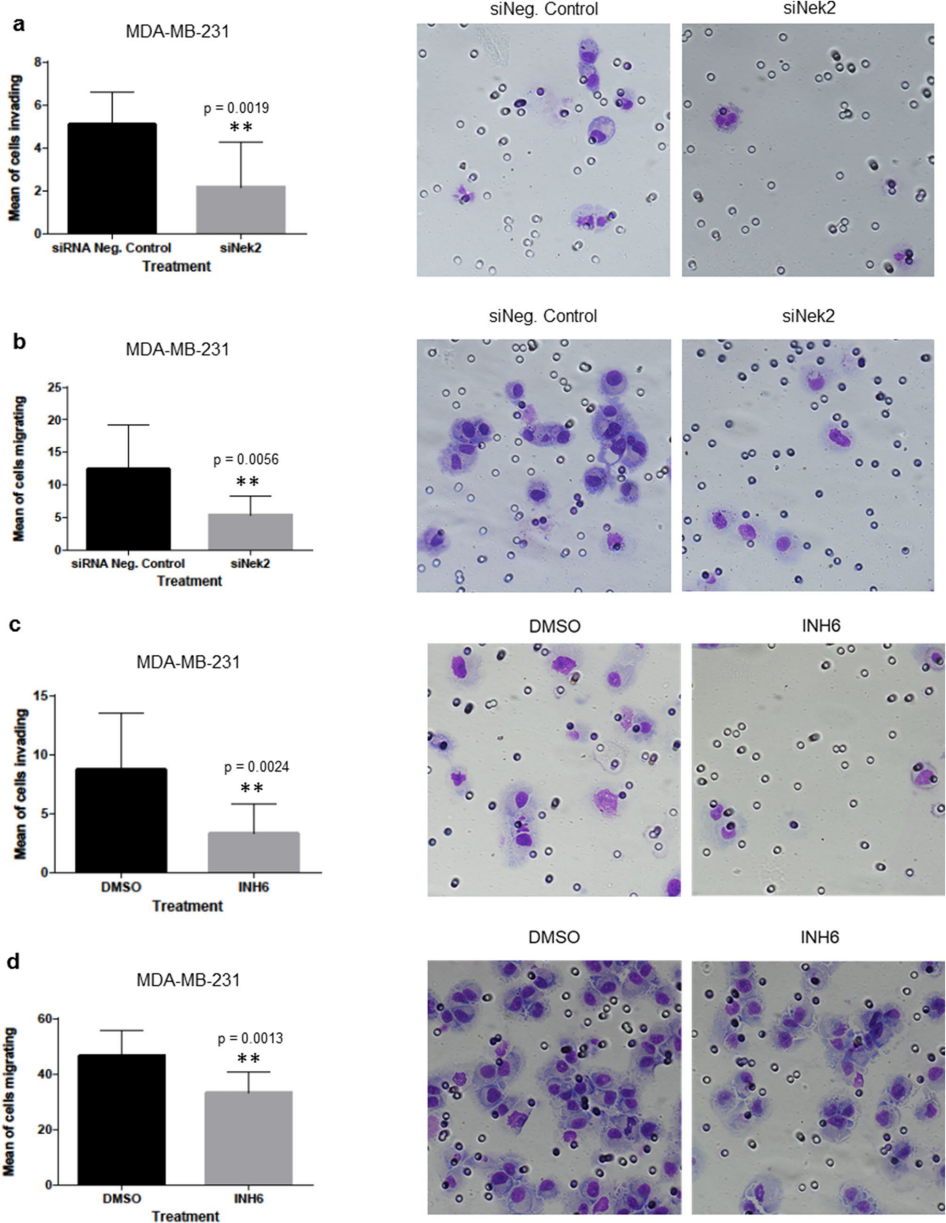

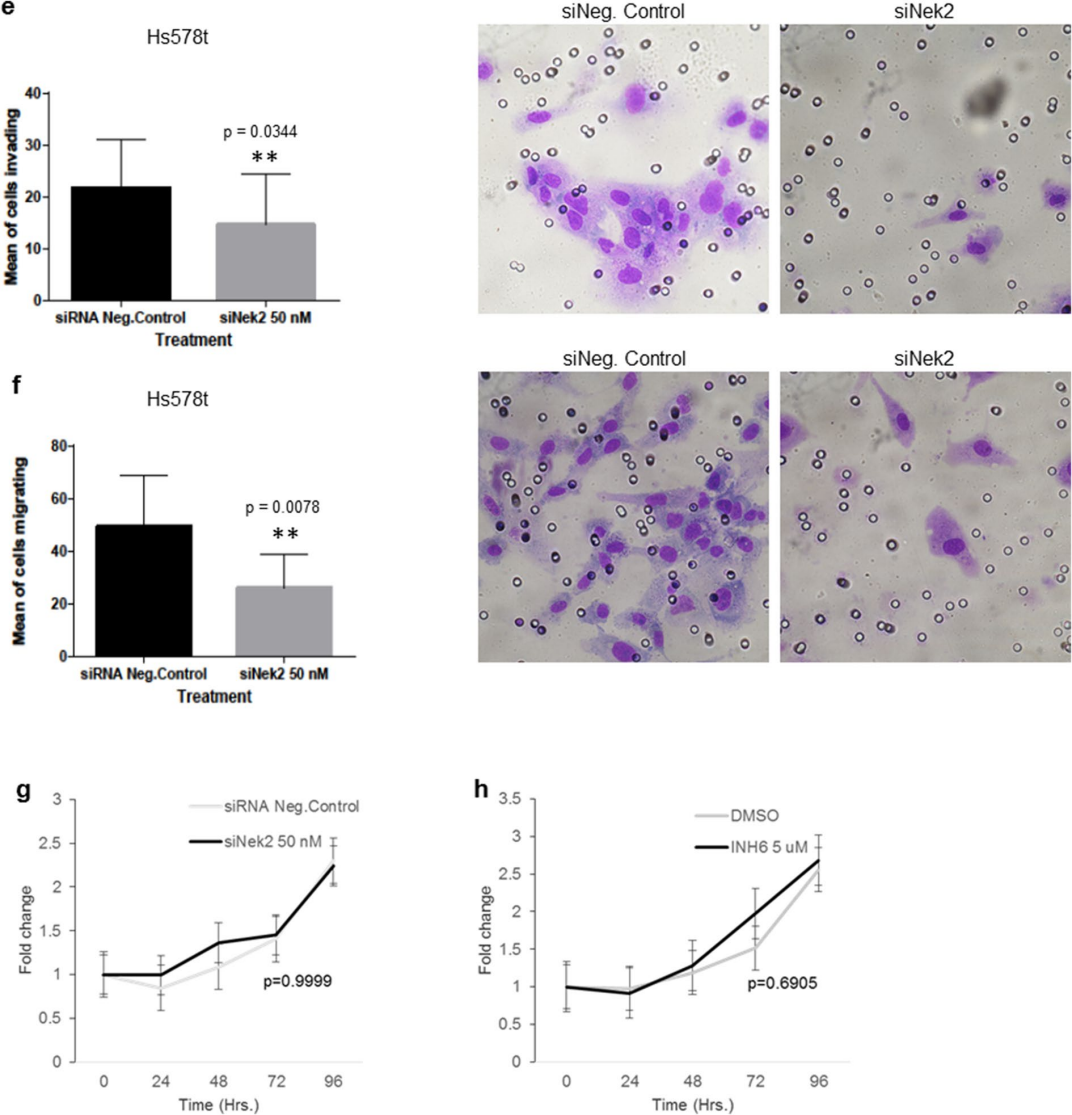
Nek2 is required for invasion and migration in triple-negative breast cancer cells. (**a–d**) Invasion and migration of MDA-MB-231 cells treated with siNek2 (**a**) and (**b**) or INH6 (c) and (**d**). (**e–f**) Invasion and migration of Hs578t treated with siNek2. (**g,h**) CCK8 cell proliferation assay was assessed in MDA-MB-231 using siNek2 (**g**) or INH6 (**h**). Data represent mean fold change ± SEM of three independent experiments.

We determined the role of Nek2 in promoting EMT in MCF10A cells. Vimentin and Slug protein levels were higher in the MCF10A/GFP-Nek2 vs control, whereas E-cadherin epithelial marker expression was reduced (Fig. 3a), indicative of EMT. Cell spreading correlated with the activation of Akt and FAK, two major regulators of cell attachment (Fig. 3b). The phosphorylation of β-catenin serves as an important control since it is a known target of Nek2 to trigger centrosome separation^82^, in addition to the STRING analysis shown in Supp. Fig. 3. The phosphorylation of β-catenin was increased upon Nek2 overexpression (Fig. 3c). To investigate if the Nek2-induced phosphorylation of β-catenin is dependent on GSK3β, we used an inhibitor specific to that molecule (Fig. 3 d). That inhibition abrogated Nek2 phosphorylation of β-catenin, suggesting that Nek2 requires GSK3 activity to phosphorylate β-catenin. This new evidence supports a putative role for Nek2 in promoting rapid cell spreading and EMT.

### Nek2 maintains the epithelial-to-mesenchymal transition in triple-negative breast cancer cells

To investigate if the biological silencing or the inactivation of Nek2 activity can reverse the mesenchymal state of breast cancer cells, we used Hs578t and MDA-MB-231, which are triple-negative breast cancer cells with mesenchymal charachteristics^72^. There are major differences between the two cell lines since Hs578t was isolated from a primary tumor and MDA-MB-231 from the ascites fluid of a breast cancer patient^72^. To accomplish this, we used two different approaches: Nek2 silencing through siRNA transient knockdown (Fig. 4) and its chemical inhibition with INH-6^49^ that prevents phosphorylation of Hec1 by Nek2, an event required for activation of the spindle assembly checkpoint (Supp. Fig. 6). In MDA-MB-231 cells Nek2 siRNA increased E-cadherin protein levels, while vimentin protein expression remained elevated (Fig. 4a). The increase in levels of E-cadherin was recapitulated with INH6 in MDA-MB-231 cells (Supp. Fig. 6a). Zeb1 levels decreased in Nek2 siRNA treated cells, but Slug only decreased in Hs578t cells (Fig. 4a,b). We could not detect E-cadherin in Hs578t cells by Western blots. Secondary anti-rabbit and mouse Alexa Fluor antibodies were used alone as our immunofluorescence controls (Fig. 4c). Immunofluorescence in cells co-immunostained for E-cadherin and Vimentin showed modest increases in E-cadherin in both MDA-MB-231 and Hs578 cells (Fig. 4 d,e). However, we observed increases in Vimentin levels in MDA-MB-231 cells and decreases in Hs578t cells. The presence of Vimentin and E-cadherin in the same cells is suggestive of hybrid EMT states within these cells. Our immunofluorescence experiments in MDA-MB-231 cells treated with INH6 indicated that in, E-cadherin and vimentin protein expression decreased (Supp. Fig. 5b). Since Slug and Zeb1 were downregulated, we used siRNA silencing to downregulate either transcription factor in MDA-MB-231 (Fig. 4d) and Hs578t cells (Fig. 4e). The silencing of Slug did not change levels of E-cadherin in MDA-MB-231 cells but increased its levels in Hs578t cells. Vimentin levels were not changed when Slug was downregulated in MDA-MB-231, but in Hs578t both the silencing of Slug and Zeb1 decreased its levels.

**Figure 6:**
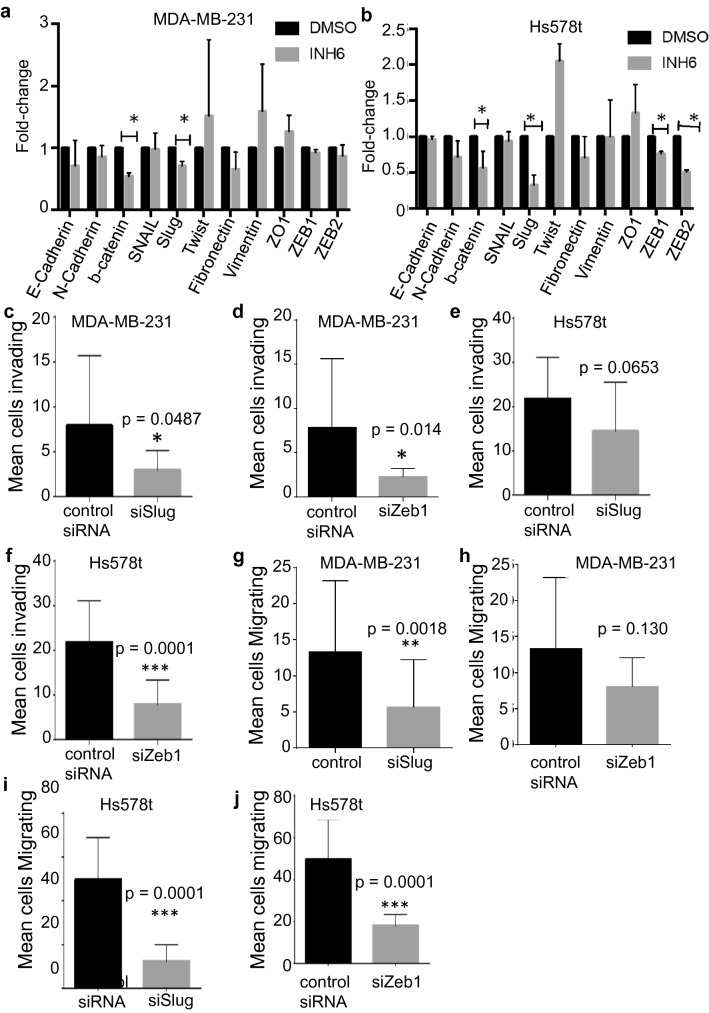
Nek2 induces mRNA level changes in EMT transcription factors. (**a,b**) qPCR analysis of EMT genes in MDA-231 and Hs578t cells. (**c–f**) Invasion of MDA-MB-231 or Hs578t treated with siSlug or siZeb1. (**g–j**) Migration of MDA-MB-231 or Hs578t treated with siSlug or siZeb1.

### Nek2 is required for the invasion and migration of triple-negative breast cancer cells

Having observed that Nek2 regulates important markers of the EMT pathway, we downregulated Nek2 to evaluate its role in invasion and migration. Both Nek2 siRNA and INH6 significantly reduced the invasion (Fig. 5a,c) and migration (Fig. 5b,d) of MDA-MB-231 compared with control. Similar results were seen with Nek2 siRNA in Hs578t cells (Fig. 5e,f). Suppression of invasion and migration may be secondary to loss of proliferation/viability; however, our CCK-8 assay experiments showed no significant differences in proliferation/viability of MDA-MB-231 cells upon Nek2 depletion (Fig. 5g,h).

### Slug and Zeb1 are involved in the invasion and migration of triple-negative breast cancer cells

Figure 4 and Suppl. Fig. 6 indicated downregulation of Slug and Zeb1 protein levels. To find additional mechanisms driving EMT, we screened several genes coding for EMT structural proteins, as well as EMT transcription factors by qRT-PCR. In MDA-MB-231 cells, Nek2 inhibition by INH6 reduced the β-catenin and Slug mRNA levels compared with control (Fig. 6a). In Hs578t, Nek2 inhibition resulted not only in reduced mRNA levels of β-catenin and Slug but also of the Zeb1 and Zeb2 EMT transcription factors (Fig. 6b). In MDA-MB-231 cells, Slug siRNA significantly reduced invasion (Fig. 6c) and migration (Fig. 6g), while Zeb1 siRNA reduced invasion (Fig. 6d) without significantly affecting migration (Fig. 6h). In Hs578t cells, Slug siRNA significantly decreased cell migration (Fig. 6i) without significantly affecting invasion (Fig. 6e), whereas Zeb1 siRNA significantly reduced both invasion (Fig. 6f) and migration (Fig. 6j) relative to control.

## Discussion

We report that the overexpression of Nek2 in the MCF10A non-transformed mammary epithelial cell line is sufficient to initiate CA, binucleation, and micronucleation and increase the percentage of cells with extra chromosomes, all of which are landmarks of CIN. The pattern of chromosome losses and sub-tetraploid gains is consistent with CA gradually inducing aneuploidy^21,79^, and not with a cytokinesis failure model leading to the propagation of the binucleated cells. We propose that if any tetraploidy was induced by cytokinesis defects and binucleation triggered by Nek2, they would be selected against as described previously^20^; consistent with published data showing that aneuploidy results in proliferation disadvantages^83^. Such manifestations of Nek2-driven CIN are in agreement with previous findings from breast cancer cell lines^25,26^ and from our work showing that Nek2 is critical to sustaining CA/CIN in Her2 + cells, in mammary epithelial cells overexpressing the E2F transcriptional activators, H-Ras^G12V^ or H-Ras^G12V^, or c-Myc^22,23,36^. Nevertheless, Nek2 was unable to initiate tumorigenesis in MCF10A cells with or without functional p53, suggesting that Nek2 is not an oncogene. This is consistent with the absence of spontaneous mammary tumors in mice overexpressing Plk4 mitotic kinase, where CA was ubiquitous^38^. It is also consistent with our data showing that despite Nek2 rescuing back CA in Her2 + breast cancer cells stably silenced for E2F3, Nek2 did not influence tumor growth^76^. However, Nek2 can help sustain tumorigenesis, illustrated by observations that Nek2 depletion in mouse models significantly reduces mammary tumor growth^42,45^.

Several studies have shown the relation between EMT biomarkers and the induction of CIN. For example, Comaills and colleagues showed that TGF-β or SNAIL induced EMT leads to mitotic defects and aneuploidy through the suppression of LaminB1 and that the genomic abnormalities persisted CTC from metastatic breast cancer patients^84^. A recent study by Khot et al. found that the transcription factor Twist induces EMT but also induces chromosome gains and losses, as well as DNA double-strand breaks. They also found that Twist down-modulates cell cycle checkpoint factors important in the regulation of CIN including Bub, BubR1, Mad1, and Mad2^85^. However, the present studies did not attempt to establish a relationship between EMT drivers and CA/CIN.

The present study identified a novel function of Nek2 that will undoubtedly advance our understanding of its ever-growing importance in cancer biology. The involvement of Nek2 in metastasis has been suggested by studies performed in *Drosophila* and breast cancer models^42,45,52^. In our model, Nek2 overexpression in non-transformed MCF10A mammary epithelial cells accelerated cell spreading and induced EMT through enhancing expression of mesenchymal marker vimentin, as well as reducing expression of the epithelial protein E-cadherin. On the other hand, in MDA-MB-231 invasive breast cancer cells silencing or chemical inhibition of Nek2 had the opposite effect of increasing E-cadherin protein expression.

How can a mitotic kinase such as Nek2 trigger cell spreading and EMT? One potential mechanism may involve the functional interactions between Nek2 and Akt, FAK, or β-catenin pathways, all known to regulate these abnormal cellular processes, and which we have shown are phosphorylated upon GFP-Nek2 overexpression in MCF10A cells. Previous reports have shown the activation of Akt and β-catenin by Nek2^47,53,55,86^, supporting the hypothesis of cross-talk between centrosomal and cytosolic signaling pathways. Our observations indicate that overexpressed GFP-Nek2 resides mostly at the centrosomes, organelles that comprise hundreds of proteins^87^. Nek2 phosphorylation of β-catenin within centrosomes contributes to the splitting of centrosomes at G2 phase^55^, but also interacts with proteins from the Wnt pathway that resides in the centrosome (adenomatous polyposis coli, Axin, and GSK3β) to signal mitotic progression^86^. Our data presented in Fig. 3 indicates that GSK3β is responsible for most of the β-catenin phosphorylation in MCF10A cells overexpressing GFP-Nek2, suggesting that perhaps Nek2 requires this kinase to fully phosphorylate β–catenin. It is conceivable that overabundant GFP-Nek2 interacts with and activates the above-mentioned centrosomal proteins that shuttle between the microtubule and actin cytoskeleton and the cytoplasm, resulting in dynamic structural changes; additionally, once the nuclear membrane breaks down in preparation for mitosis, GFP-Nek2 can untimely phosphorylate these proteins. Thus, one potential mechanism explaining our results is the phosphorylation of β-catenin by Nek2. It has been previously reported that cytoplasmic and nuclear β-catenin can bind the vimentin promoter and induce its activity^88^. There was a discrepancy regarding β-catenin levels between MDA-MB-231 and Hs578t cell lines, where its overall levels were reduced in the former and membrane-bound β-catenin enhanced in the latter. The isolation sites may explain some of the differences; MDA-MB-231 was derived from an adenocarcinoma isolated by pleural effusion, while Hs578t was isolated from a primary invasive ductal carcinoma of a TNBC patient^72^. Therefore, the involvement of β-catenin will be explored in future studies.

We find that unregulated Nek2 changes the levels of several EMT transcription factors (Slug and Zeb1). The overexpression of Nek2 in MCF10A cells led to increased expression of Slug, a member of the Snail superfamily of transcription factors. We also show that the chemical inhibition or downregulation of Nek2 in MDA-MB-231 cells did not significantly downregulate Slug protein. Nek2 inhibition resulted in lower levels of Slug mRNA, while its inhibition in Hs578t cells results in lower levels of Slug, Zeb1, and Zeb2. Zeb1 protein was consistently downregulated when Nek2 is silent in these cell lines. It is known that one of the signaling pathways sustaining EMT often converges onto transcription factors including Snail, Slug, Twist, Zeb1, and Zeb2^89,90^. These transcriptional factors are involved in the suppression of genes that encode cadherins, claudins, occludins, plakophilins, MUC1, and cytokeratins^91^. High expression of Slug correlates with reduced E-cadherin, high histologic grade, lymph node metastasis, postoperative relapse, and decreased survival in different cancers^92–94^. A recent study investigating the expression of EMT transcription factors in primary cancer cell lines from breast, colon, ovarian, and head and neck squamous carcinoma^95^ demonstrated high in vivo expression levels of Zeb1 and Zeb2 in ovarian (SKOV3) and in vitro breast cancer cells (MDA-MB-231). Silencing of Slug or Zeb2 can significantly suppress invasion and migration in both cell lines and silencing of either transcription factor decrease levels of Vimentin. Thus, our data is consistent with previous studies pointing out that Slug and Zeb1 are important regulators of EMT; the novelty is that Nek2 can de-regulate these transcription factors.

Cell invasion is a highly-relevant early step in the metastatic cascade. Nek2 could also drive invasion through CA; where CA alters microtubule organization, thus providing additional cytoskeletal advantages and modifying cell polarization towards a migratory/invasive profile^96^. There is precedent for this mechanism, since the induction of Plk4, a regulator of centriole duplication, leads to CA and subsequent cell invasion in MCF10A cells^97^. Likewise, our group previously showed that overexpression of GFP-Nek2 rescues back CA/CIN in Her2 + breast cancer cells that were silenced for E2F3 and also induces invasive protrusions^22^. Neither of the aforementioned studies demonstrated a direct role for CA or Nek2 in EMT.

In summary, we observed a significant reduction in both migration and invasion of breast cancer cells depleted of Nek2 through siRNA or INH6. We noticed similar outcomes with siRNA against Slug or Zeb1. This is the first study demonstrating the involvement of Nek2 as a regulator of Slug, and Zeb1 in migration and invasion of MDA-MB-231 and Hs578t TNBC cells. Despite showing promising preclinical results, none of the compounds with high specificity and irreversible inhibition for Nek2 is being tested in clinical trials^98^. In-depth characterization of the role of Nek2 in cell spreading, adhesion, EMT, invasion, and metastasis will help refine the therapeutic strategies targeting Nek2.

## Materials and methods

### Bioinformatic analyses

The expression of Nek2 in METABRIC was assessed with http://www.cbioportal.org/ using z-scores using a threshold of 2. Its expression in different molecular subtypes was assessed by applying the Pam50 filter and downloading the data into Microsoft Excel 97-2003 software.

### Cell culture

MCF10A (CRL-10317), MDA-MB-231 (HTB-26), and Hs578t (HTB-126) cell lines were purchased from ATCC and cultured as described^11^.

### Plasmid DNA transfections

Nek2 was subcloned into the pMONO-Hygro-GFP plasmid by the Emory DNA Custom Cloning Core Facility. Transfection of MCF10A cells was done using TransIT-2020 Transfection Reagent (Mirus MIR 5400A), and populations were selected and maintained in media containing 25 μg/mL hygromycin (Sigma G8168).

### RNA interference

MDA-MB-231 and Hs578t cells were seeded on 6 well plates, incubated, and allowed to attach overnight. After 24 h, cells were transfected using 50 nM ON-TARGET *plus* Human siRNAs from Dharmacon for either Nek2 (L-004090-00-0005), Slug/SNAI2 (L-017386-00-0005), Zeb1 (L-006564-01-0005), or silencer negative control siRNA (Invitrogen AM4611) and mixed with jetPRIME Transfection Reagent (Polyplus 114-07) in cell media according to manufacturer instructions. Transfection complex was allowed for 48 h and used for the experiments described below.

### Immunofluorescence and image acquisition

GFP-expressing MCF10A cells were plated at a density of 100,000 cells/well in 4-well slides, allowed to attach overnight and processed as described^11^. The following primary antibodies and reagents were used: from Pericentrin (Abcam Ab4448), from Cell Signaling vimentin (5741S), E-cadherin (3195S), ZO-1 (8193S), Slug (9585S), Zeb1 (3396S), β-catenin (8480S), from Santa Cruz α-tubulin (sc-32293), and Alexa Fluor-conjugated phalloidin. Alexa Fluor-conjugated antibodies (Thermo-Fisher) were used as secondary antibodies. As a counterstaining, DAPI (1 mg/mL) was applied. All fixed samples were mounted in Fluorogel mounting medium. Images were taken using the following microscopes from the Winship Cancer Institute ICI Core: Zeiss Axioplan 2 widefield, Zeiss LSM 510 META confocal, Leica SP8 confocal, and DeltaVision OMX Blaze super-resolution. For the time-lapse microscopy, proliferating cells were plated on an eight-well chambered #1.5 German coverglass system. Live cells were imaged at 20 × on the Perkin Elmer Ultra View Spinning Disk microscope at 37 °C and 5% CO_2_. Images were captured every five minutes for 72 h and compiled into movies for analysis. All image capture and analysis were done using the Imaris Analysis Software. Images for the inhibition with INH6 were collected using similar microscopes at Moffitt Cancer Center. Binucleated cells (α-tubulin) cell with micronuclei (DAPI) and cells ≥ 3 centrosomes (pericentrin) were counted and expressed as a percentage of the total number of cells.

### Western blotting

Western blots were done using our lab protocols^11,22,23,36,76,99^. Previous to development of films the membranes were cut in order to be able to probe with different antibodies. The following primary antibodies were used: from BD Biosciences Nek2 (610594), FAK (3285S), GFP (Abcam Ab290), from Cell Signaling p-Akt S473 (4060S), Akt (9272S), p-FAK Tyr397 (3283S), E-cadherin (3195S), ZO-1 (8193S), β-catenin (8480S), slug (9585S), Zeb1 (3396S), and vimentin (5741S). β-actin was used as a loading control (Santa Cruz sc-47778). The X-ray fims were also cut in quarters order to save money, and thus the films presented in Supplementary figures are of irregular shapes. HRP-conjugated secondary antibodies were used (Santa Cruz sc-2004 and sc-2005). Signals were detected by using a Lumigen TMA-6 reagent.

### Chromosome spreads

Chromosome spreads were obtained as described in our publications^21^.

### 3D matrigel culture and measurement of acini volume

Three-dimensional cultures were done as described in our publications^76^. Alexa Fluor 555-conjugated phalloidin was incubated overnight at room temperature, followed by DAPI counterstain. Slides were mounted and z-stack images were acquired using the Zeiss LSM 510 META confocal microscope. Organoid volumes were calculated using the Imaris software.

### Cell spreading assay

Proliferating cells were trypsinized and allowed to spread for 45 and 90 min before fixation with 4% paraformaldehyde. Fixed cells were prepared for immunofluorescence with Alexa Fluor 555-conjugated phalloidin as previously described. Images were acquired using a Leica SP8 confocal microscope. Cell spreading (phalloidin) was quantified using CellProfiler.

### Preparation of cells for xenograft model

Six to eight weeks female athymic mice Crl:NU (NCr)-*Foxn1*^*nu*^ were purchased from Charles River and treated according to the animal care guidelines specified in the protocol and approved by the Institutional Animal Care and Use Committees (IACUC) from the Winship Cancer Institute, Emory University School of Medicine. Also, our IACUC protocol was in compliance with the ARRIVE guidelines. MCF10A p53^-/-^ cells expressing either GFP or GFP-Nek2 were passaged 48 h before the implant. Cells were injected into the mammary fat pad of 9 weeks old mice as described in our publications^76^. Tumor size, both length, and width was measured starting 10 days after injection and continued every day for two weeks. Tumor mass was calculated by the formula [area (width x length) x length] 2.

### Nek2 chemical inhibition

Hec1/Nek2 Mitotic Pathway Inhibitor II, INH6 (Millipore/Sigma 373271) was used at a concentration of 5 µM for a time point of 96 h. The dose was changed after 48 h and allow for an additional 48 h upon completing 96 h for further experiments.

### RNA extraction and quantitative real-time PCR

Total RNA from each cell sample was extracted using the RNeasy Mini kit (Qiagen 1002137). The absorbance ratio at 260/280 nm of the isolated RNA samples was measured using the NanoDrop 2000c spectrophotometer (Thermo Fisher) and an aliquot of 1 μg total RNA was subjected to a reverse transcriptase reaction using the iScript cDNA Synthesis kit (BioRad 1708891). Gene expression levels were measured using RT^2^ qPCR primers assay (BioRad 1708880) using Qiagen primers for human CDH1/E-cadherin (PPH00135F-200), CDH2/N-cadherin (PPH00636F-200), CTNNB1/β-catenin (PPH00643F-200), SNAI1/Snail (PPH02459B-200), SNAI2/Slug (PPH02475A-200), TWIST1 (PPH02132A-200), FN1/Fibronectin (PPH00143B-200), VIM (PPH00417F-200), TJP1/ZO-1 (PPH09919F-200), ZEB1/Zeb1 (PPH01922A-200), ZEB2/Zeb2 (PPH09021B-200), and GAPDH (PPH00150F-200). Each primer was mixed with cDNA and iQ SYBR Green Supermix (Bio-Rad 1708880) and subjected to 39 cycles using a Realplex2 (ThermoFisher). Obtained data from three independent experiments were presented as the average. The fold change for each gene relative to the control group was determined using the 2-ΔΔCT method.

### In vitro migration and invasion assays

Invasion of MDA-MB-231 and Hs578t cells was assayed in a BD Matrigel 24-well Invasion Chamber (354480 and 354578). Briefly, 500 µL containing 5 × 10^4^ cells in media with 2% FBS were seeded in the upper compartment of the chamber, and 750 µL complete media with 10% FBS was loaded in the lower compartment of the chamber. Cells were allowed to invade for 24 h. The two compartments of this chamber are separated by Matrigel (10-µm thickness and 8-µm pore size). Uncoated membranes were used as a control for cell migration, according to the manufacturer's protocol. After 24 h the chamber was removed, and non-invading cells in the upper compartment were removed using a hyssop/cotton swab, and cells that invaded the bottom of the matrigel membrane were fixed and stained with HEMA 3 Stat Pack (Fisher 122–911). A total of 12 fields/groups (4 fields/treatment on each experiment) were counted by light microscopy. The mean of invading and migrating cells were calculated from three independent experiments.

### Cell counting kit-8

2,000 cells were seeded in 96 well plates and incubated for 24 h for treatment with siNek2 or INH6. Proliferation was measured at 0, 24, 48, 72, and 96 h after treatments using the CCK-8 (Dojindo CK04-11). Fold changes were calculated from three independent experiments.

### In silico analysis for protein–protein interaction networks

Correlations of Nek2 with important EMT pathways were done using STRING (http://www.string-db.org). The interpretation of those networks is as follows: Nodes: number of proteins in the network; Edges: number of interactions within the proteins; Node degree: average number of interactions within proteins; Clustering coefficient: denotes the tendency of the network to form clusters (the closer this value is to 1, the more likely it is to form clusters); protein–protein interaction enrichment p-value: denotes the statistical significance. Proteins are considered hubs when they overpass the average interactions (nº interactions > node degree).

### Statistical analysis

Data were reported as the means ± standard deviation. Unpaired Student *t-test* using Mann–Whitney test was applied to assess the significances between GFP control and GFP-Nek2 groups, control siRNA, and Nek2 siRNA, Slug siRNA, or Zeb1 siRNA, and also for DMSO control and INH6 using GraphPad Prism 7.0 and Microsoft Excel 97-2003 softwares. Each experiment was repeated three times. A *p*-value ≤ 0.05 was considered statistically significant.*p ≤ 0.05, **p ≤ 0.01, and ***p ≤ 0.001.

## Supplementary information

Supplementary Information.

## Data Availability

All data generated or analyzed during this study are included in this published article (and its Supplementary Information files).
