# Supplementary material for: The Nek2 centrosome-mitotic kinase contributes to the mesenchymal state, cell invasion, and migration of triple-negative breast cancer cells
Source: Sci Rep. 2021 Apr 27;11:9016. doi: 10.1038/s41598-021-88512-0 (PMC8079711; doi:10.1038/s41598-021-88512-0)

The Nek2 centrosome-mitotic kinase contributes to the mesenchymal state, cell invasion, and migration of triple-negative breast cancer cells.

Yainyrette Rivera-Rivera[1*], Mihaela Marina[2*], Shirley Jusino[1], Miyoung Lee[3], Jaleisha Vélez Velázquez[4], Camille Chardón-Colón[1], Geraldine Vargas[1], Jaya Padmanabhan[5], Srikumar P. Chellappan[5], and Harold I. Saavedra[1]

Supplementary Figure 1. Nek2 mRNA expression in different breast cancer subtypes using Oncomine

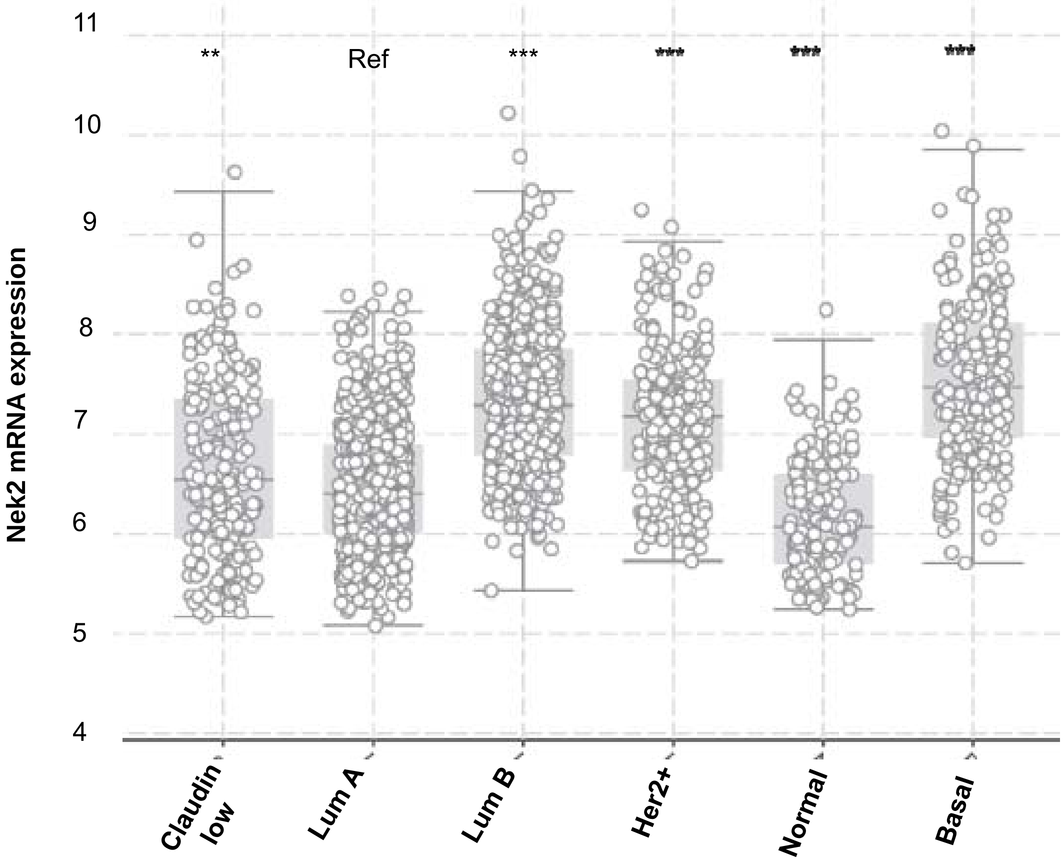

Supplementary Figure 2. Nek2 overexpression causes chromosome instability.

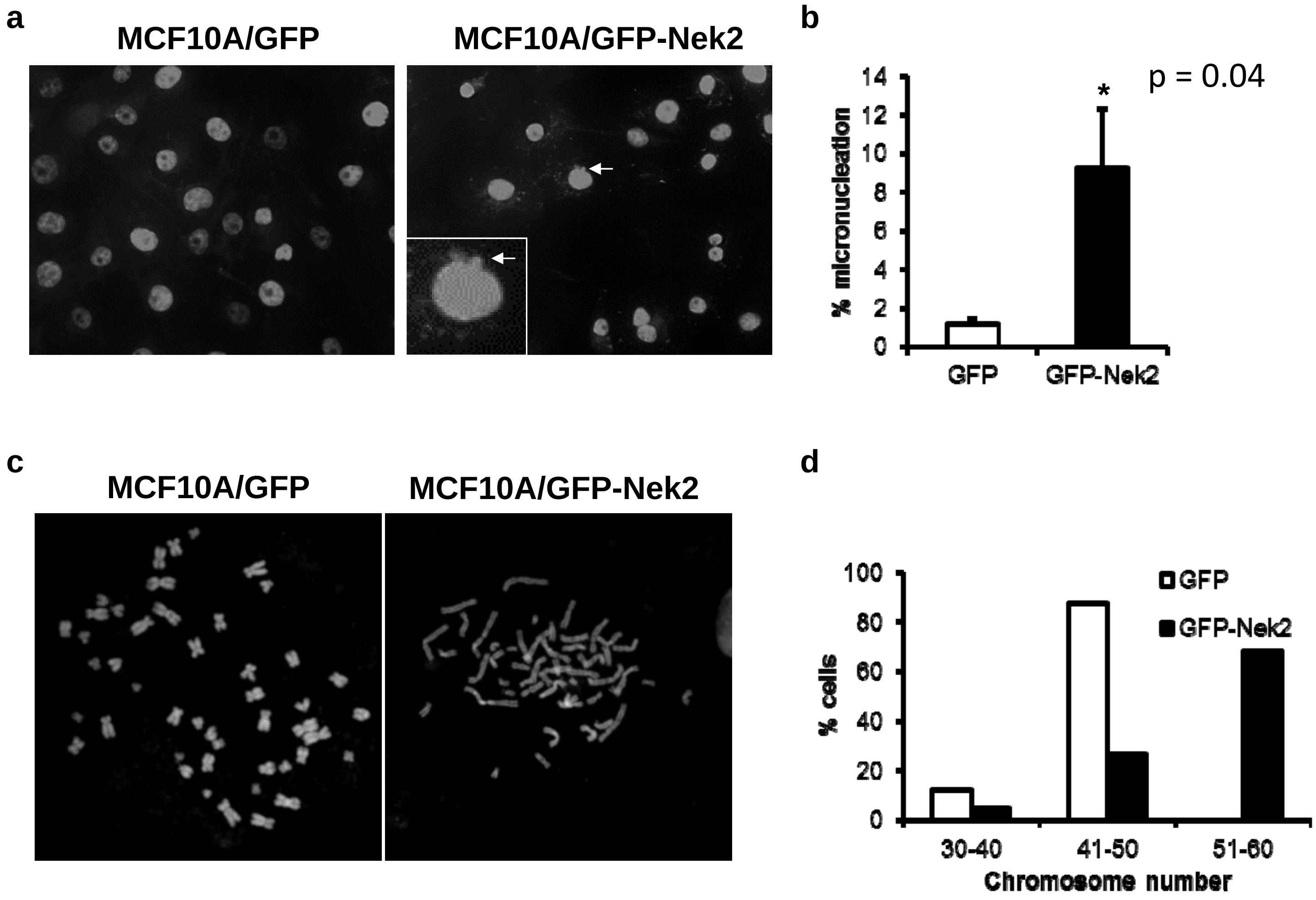

Supplementary Figure 3. Nek2 overexpression increases organoid size

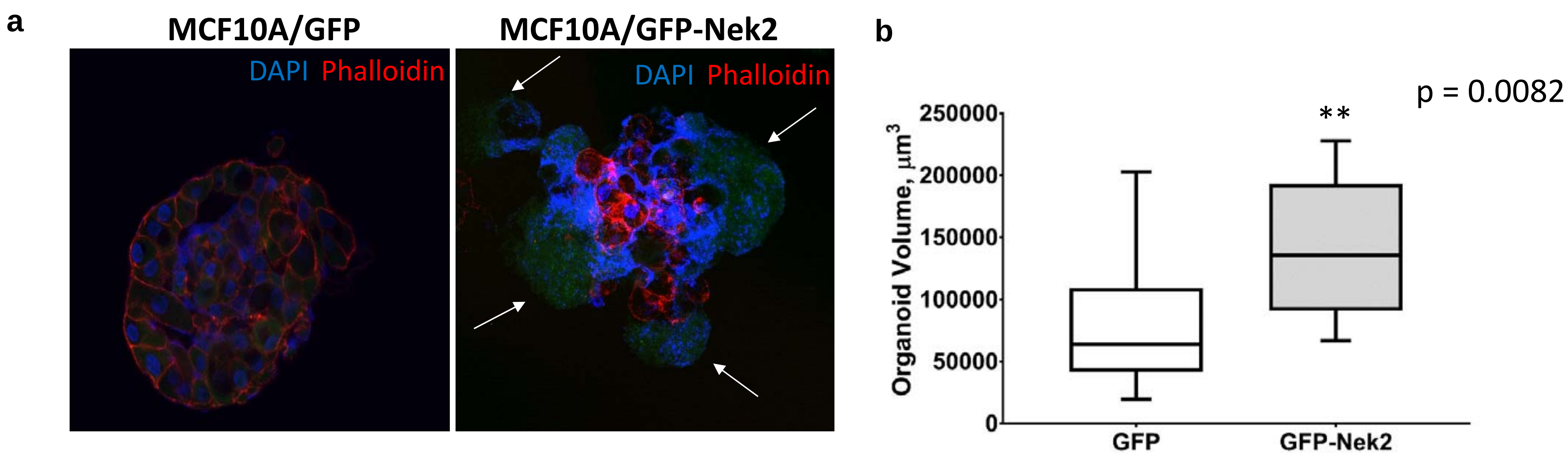

Supplementary Figure 4. Nek2 overexpression increases cell spreading

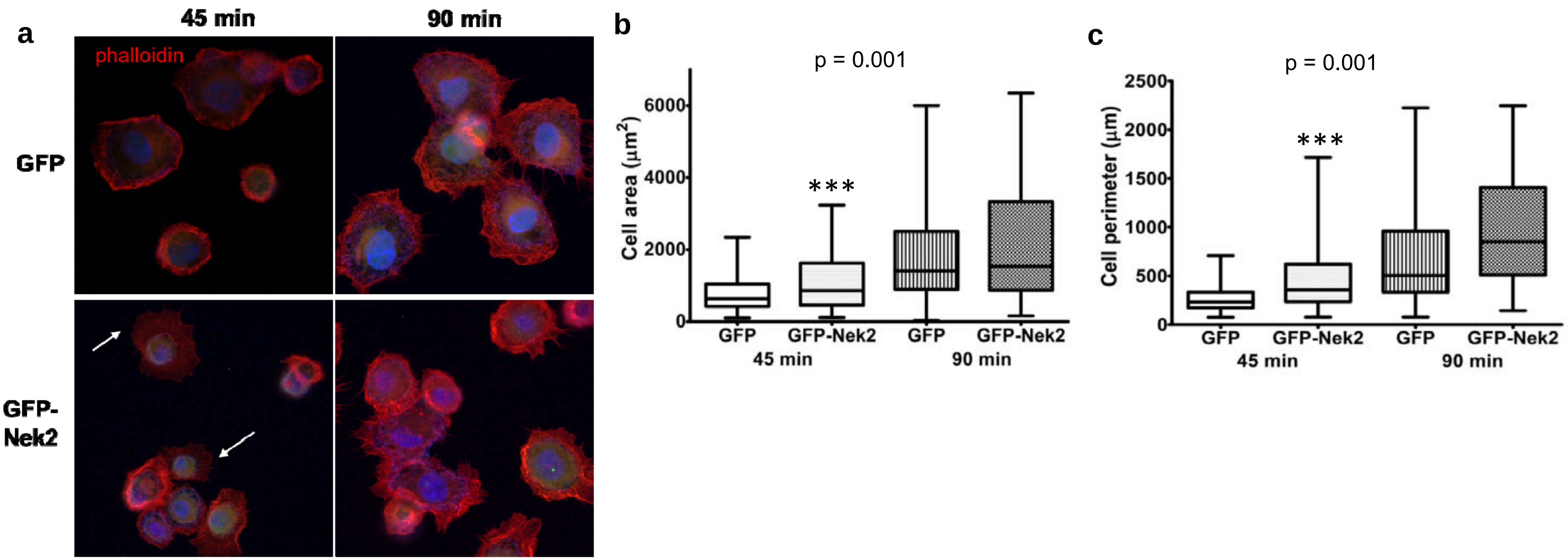

Supplementary Table 1. Gene expression of Nek2 in different molecular breast cancer subtypes

	Luminal A	Luminal B	Basal	Her2+	Normal-Like	Claudin-Low
Number of patients with the indicated subtype	679	461	199	220	140	199
Number of patients overexpressing Nek2	0	24	20	10	0	5
Percentage of patients overexpressing Nek2	0	5.2 %	10 %	4.5 %	0 %	2.5 %
Number of patients with Amplified Nek2	215	121	25	40	15	15
Percentage of patients with Amplified Nek2	31.7 %	26.2 %	12.6 %	18.2 %	10.7 %	7.5 %

Supplementary Table 2. Nek2 overexpression causes mitotic perturbations

Phenotype	%	
	MCF10A/GFP	MCF10A/GFP-Nek2
Normal division	98.67	68.06
Tripolar division	0.44	3.47
Binucleation	0.44	12.5
Cytokinesis defects	0	10.42
Cell death	0.44	5.56

Supplementary Figure 5. String analysis predicted interactions between Nek2 and EMT proteins.

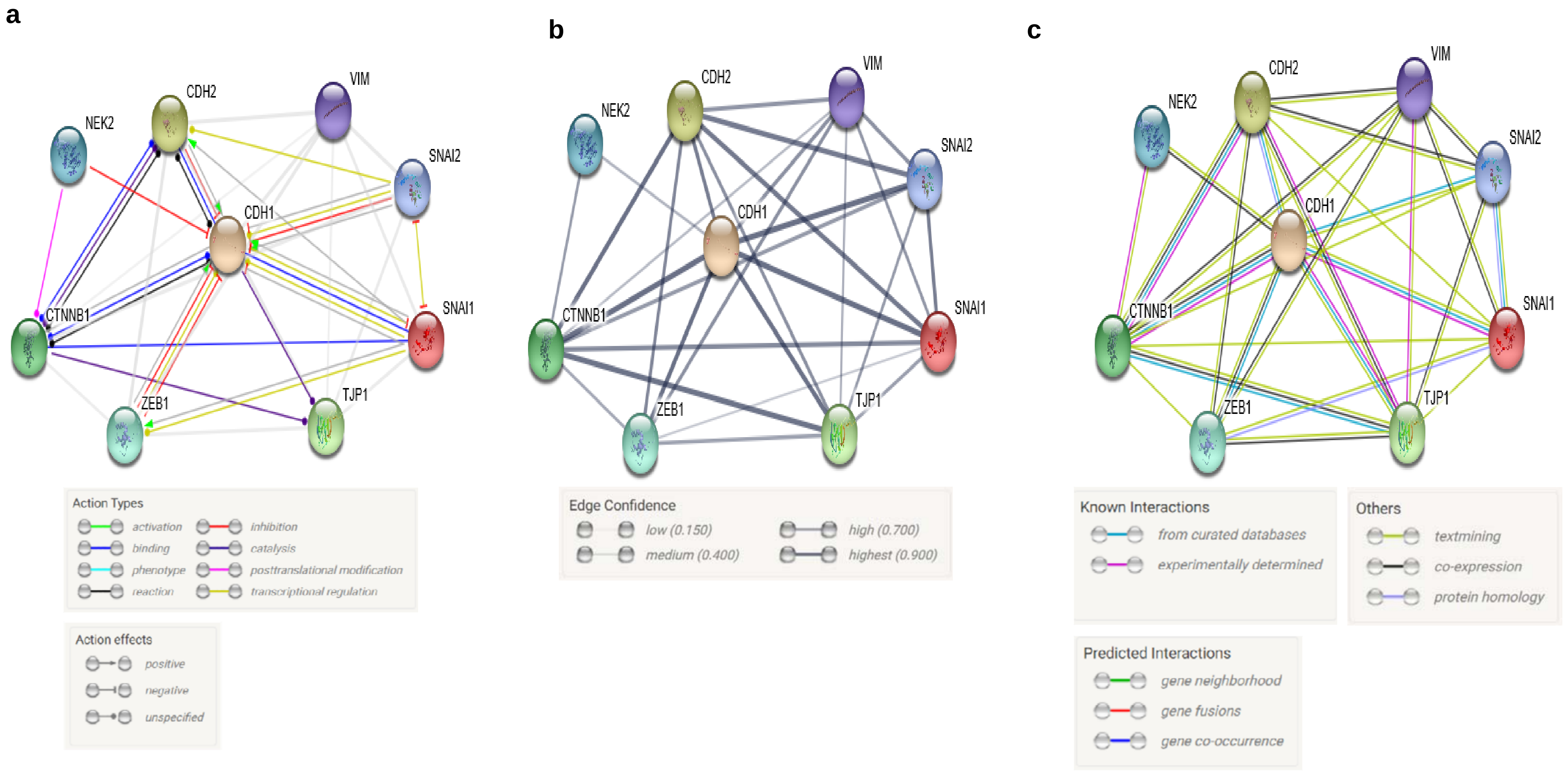

Supplementary Figure 6. The INH-6 Nek2 inhibitor suppresses EMT markers

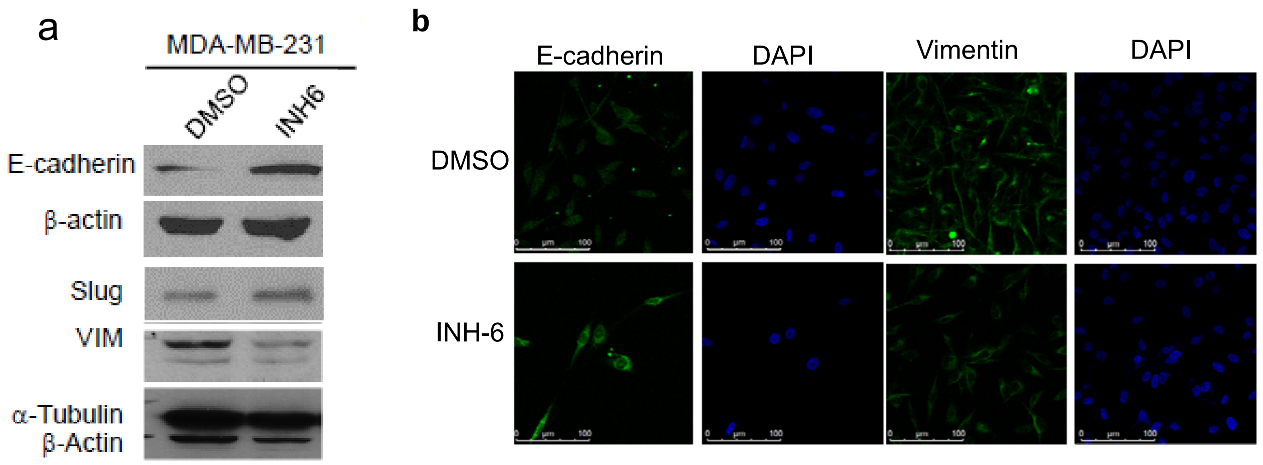

Original Western Blots for Fig. 1a.

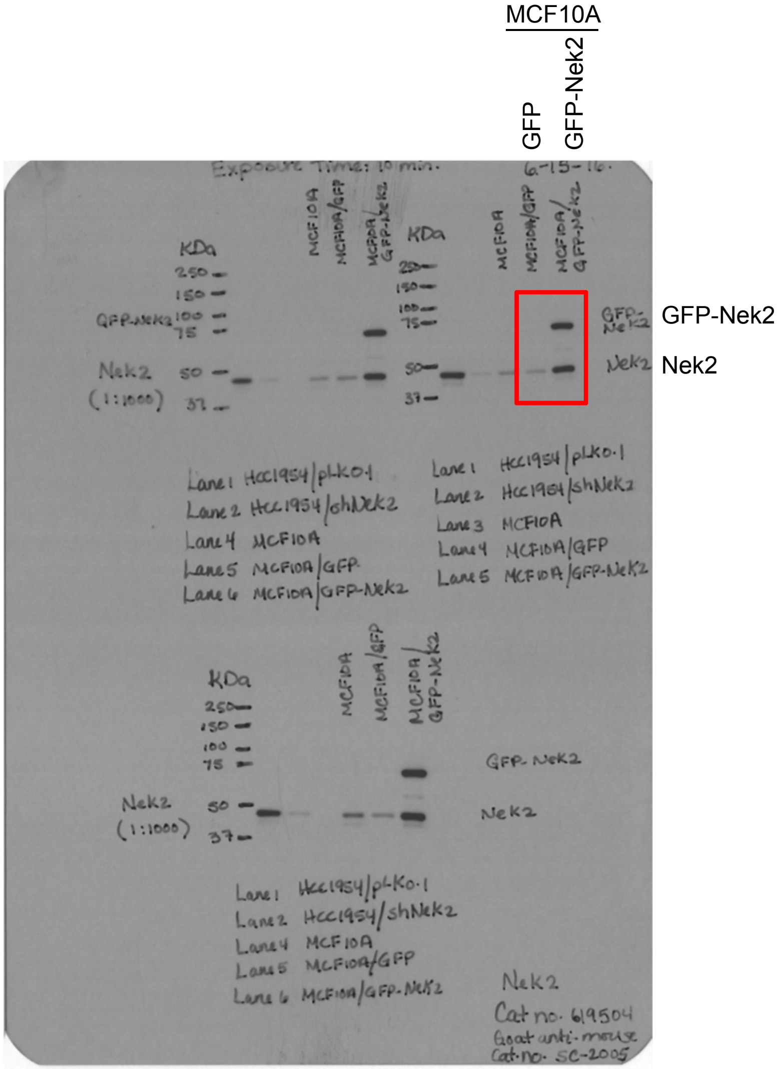

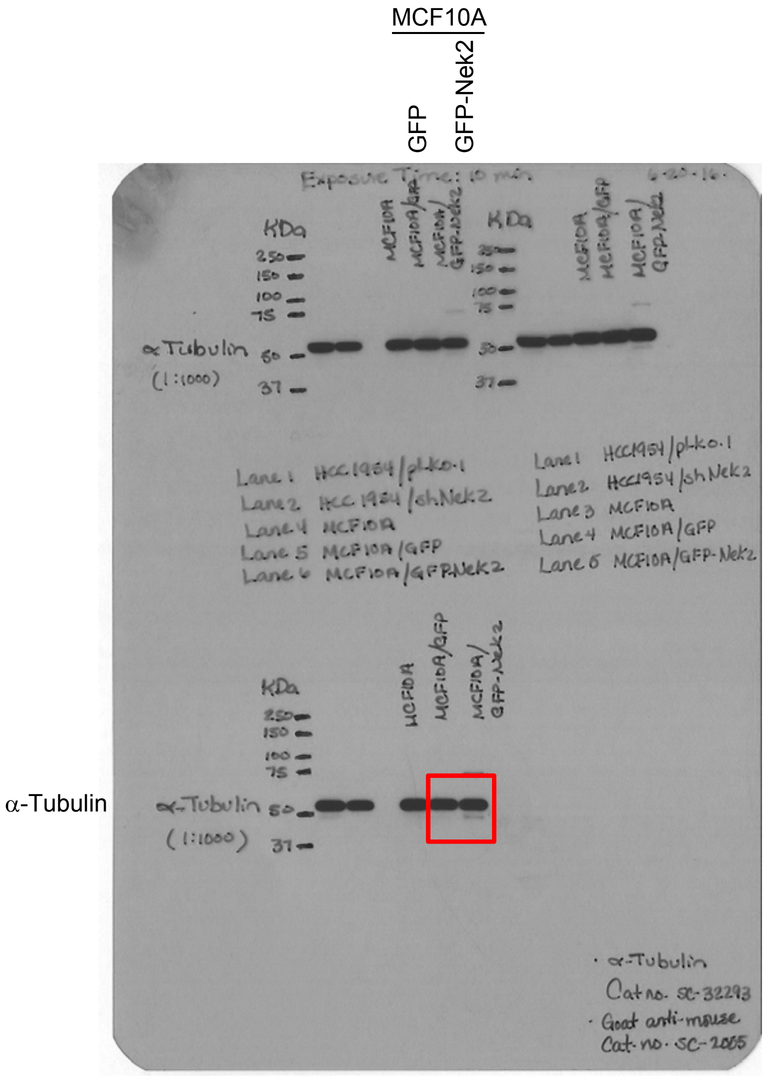

Original Western Blots for Fig. 2b and c

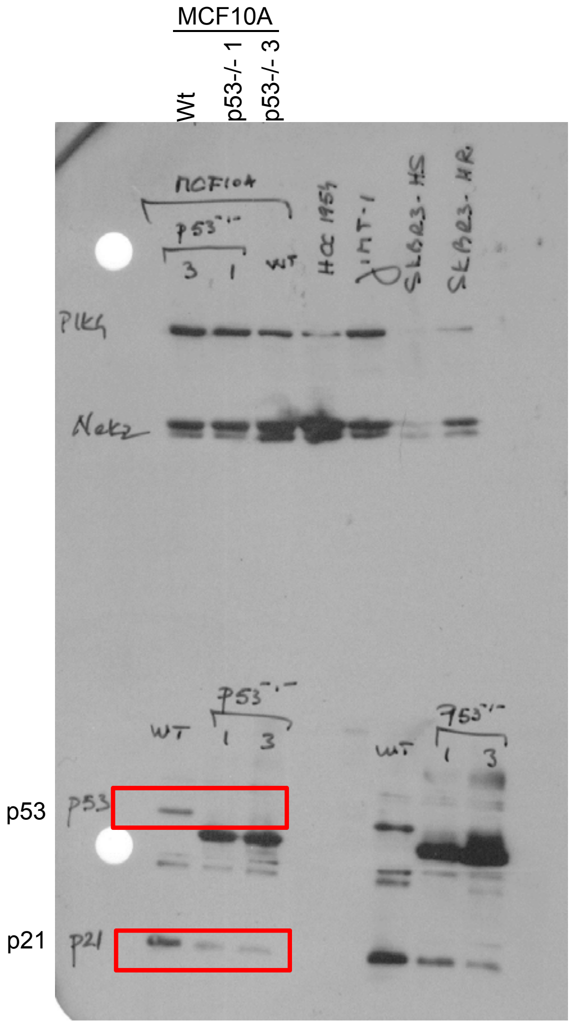

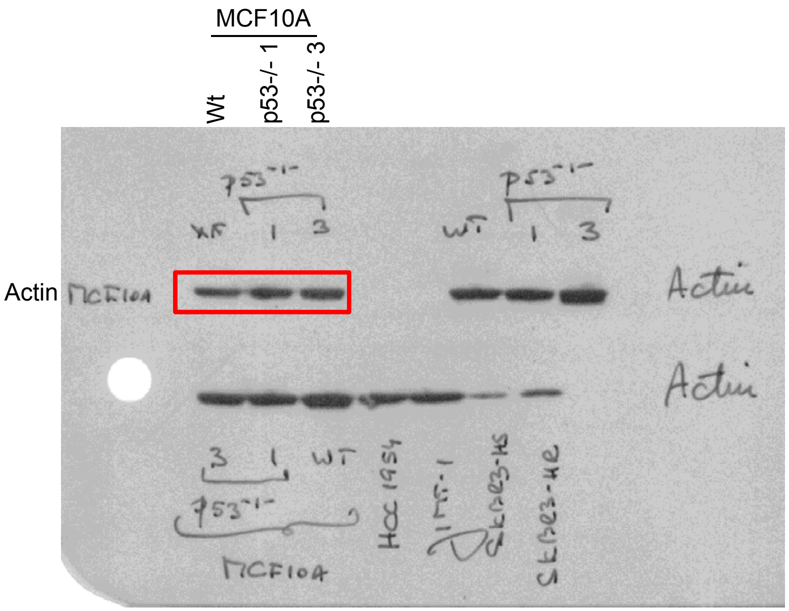

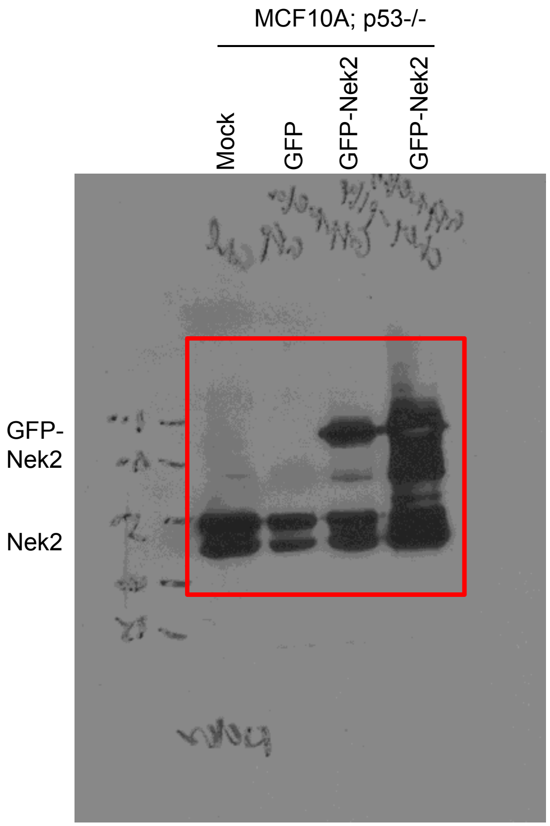

Original Western Blots for Fig. 3a

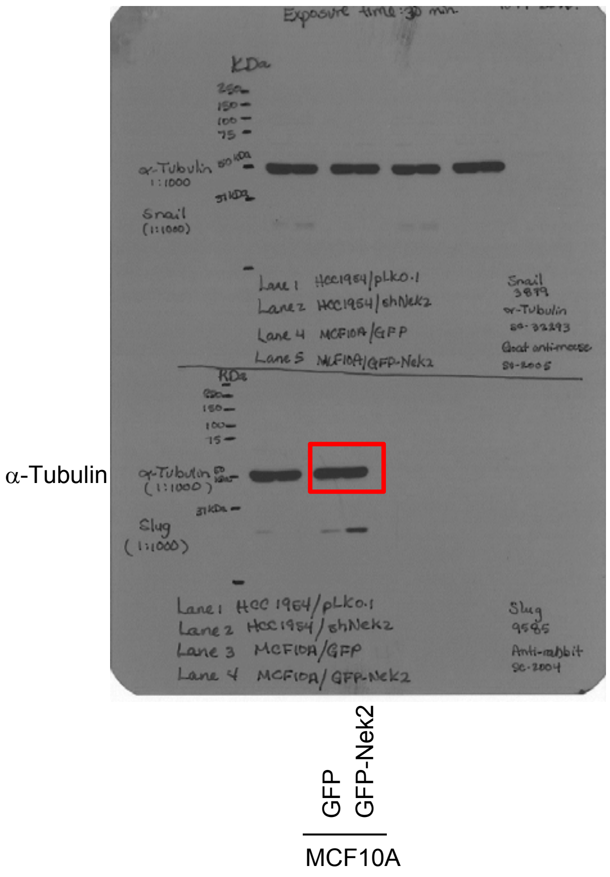

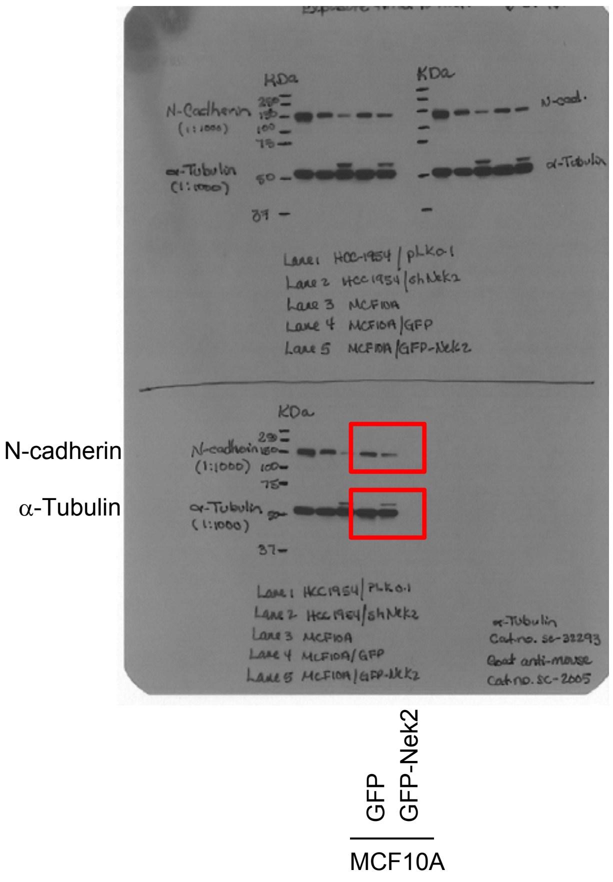

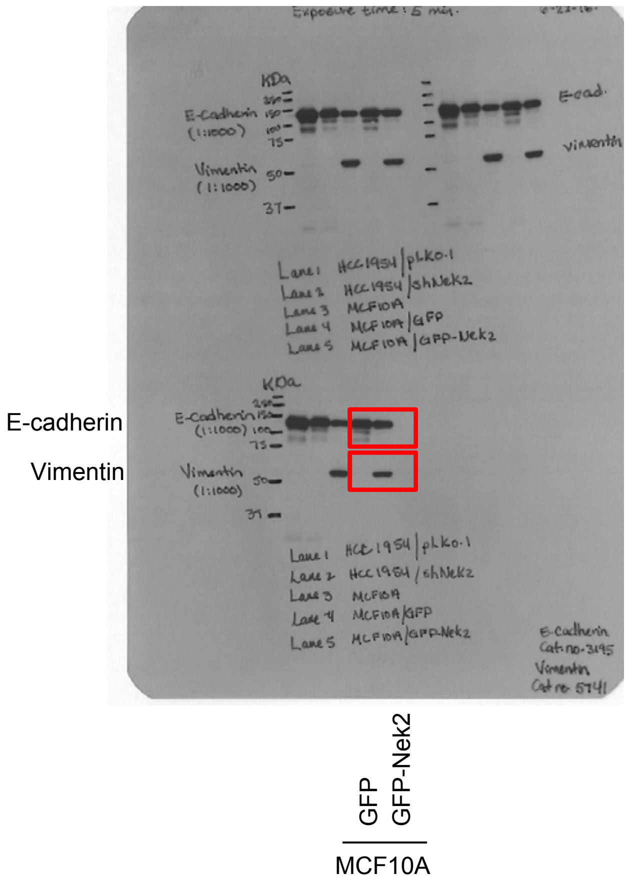

Original Western Blots for Fig. 3b

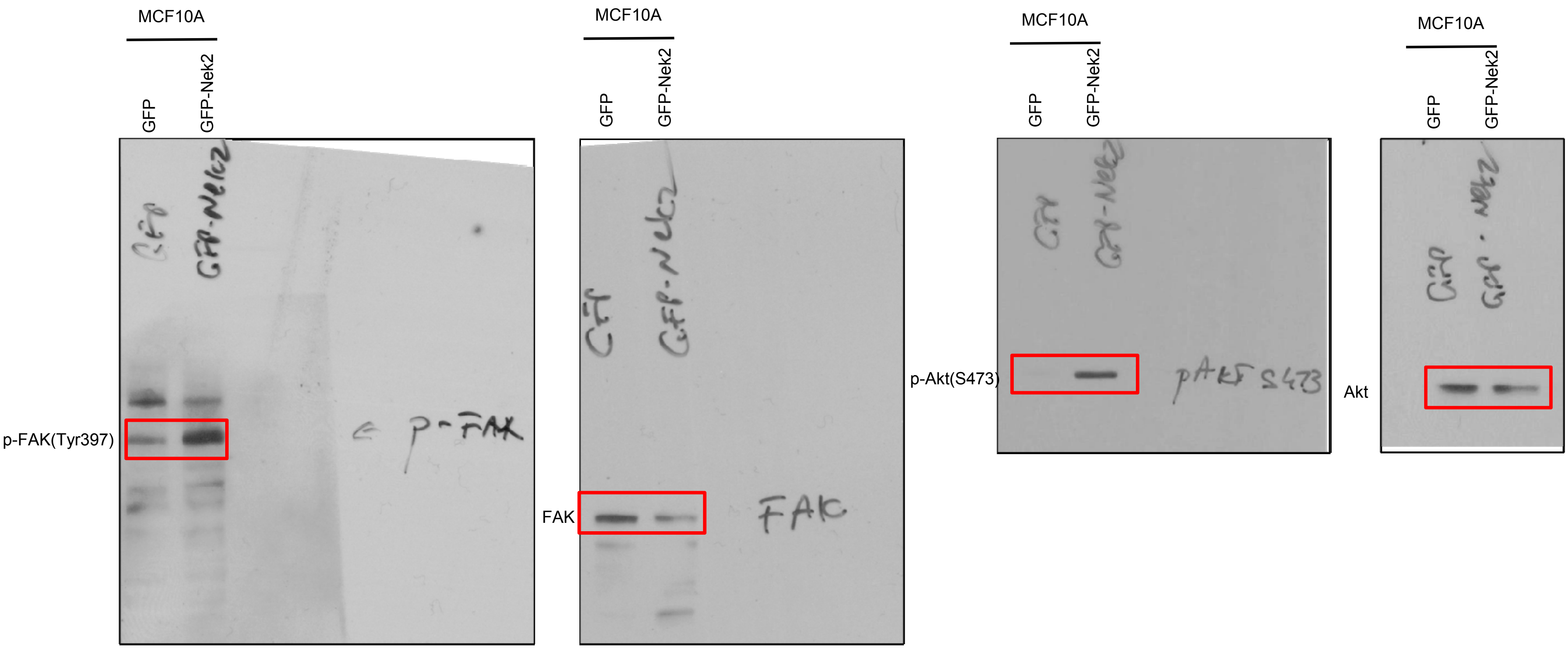

Original Western Blots for Fig. 3c

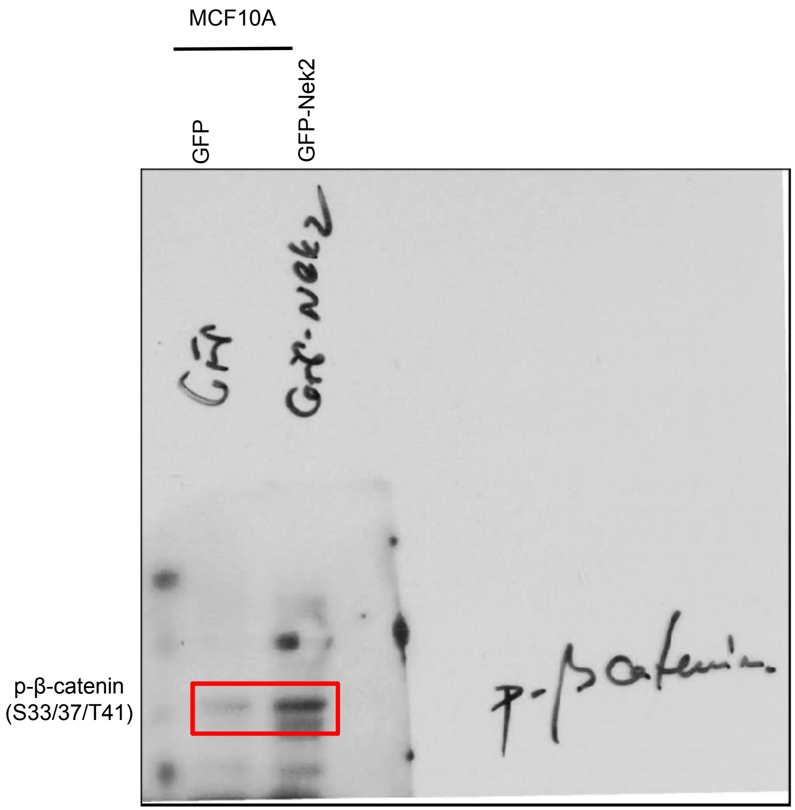

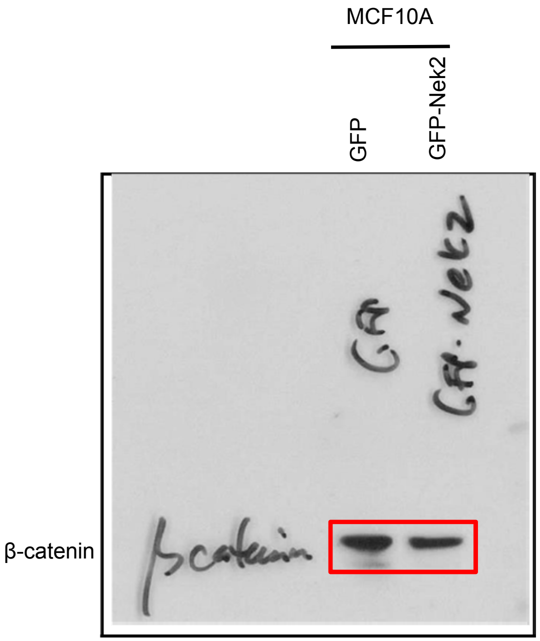

Original Western Blots for Fig. 3d

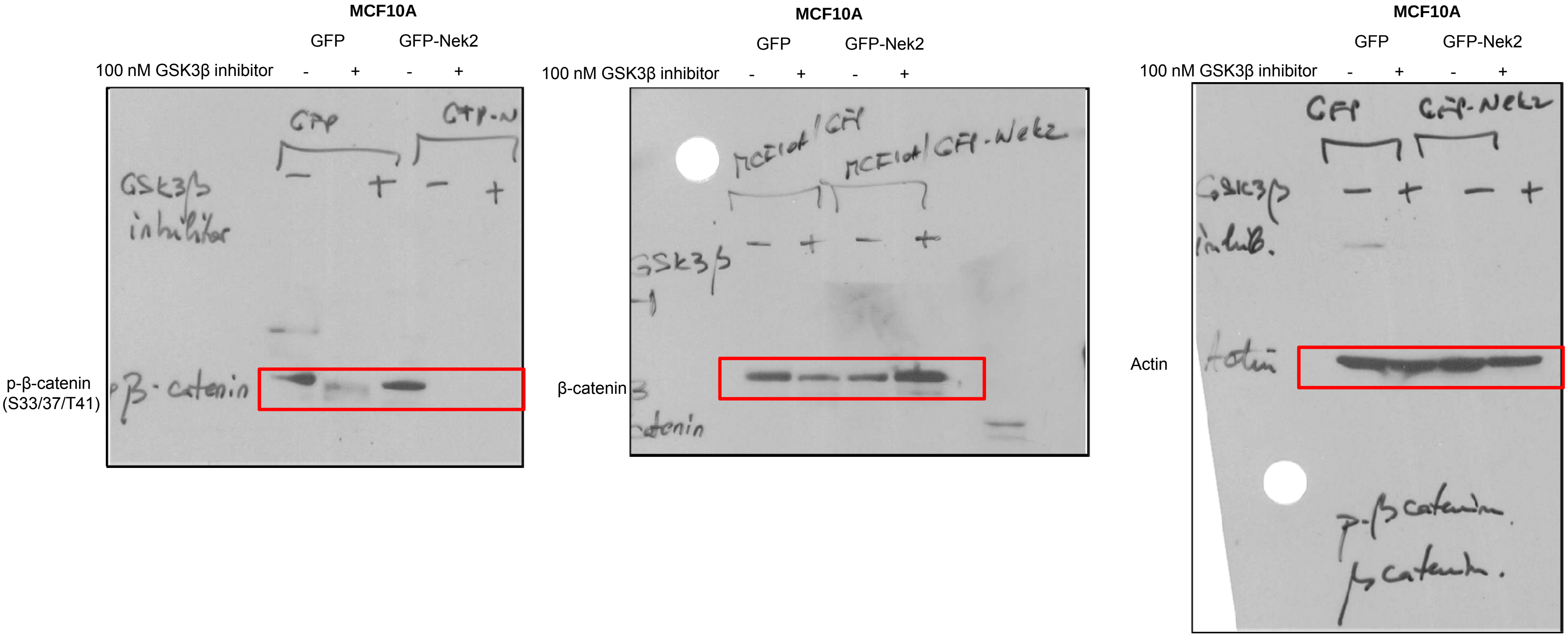

Original Western Blots for Fig. 4 a-b

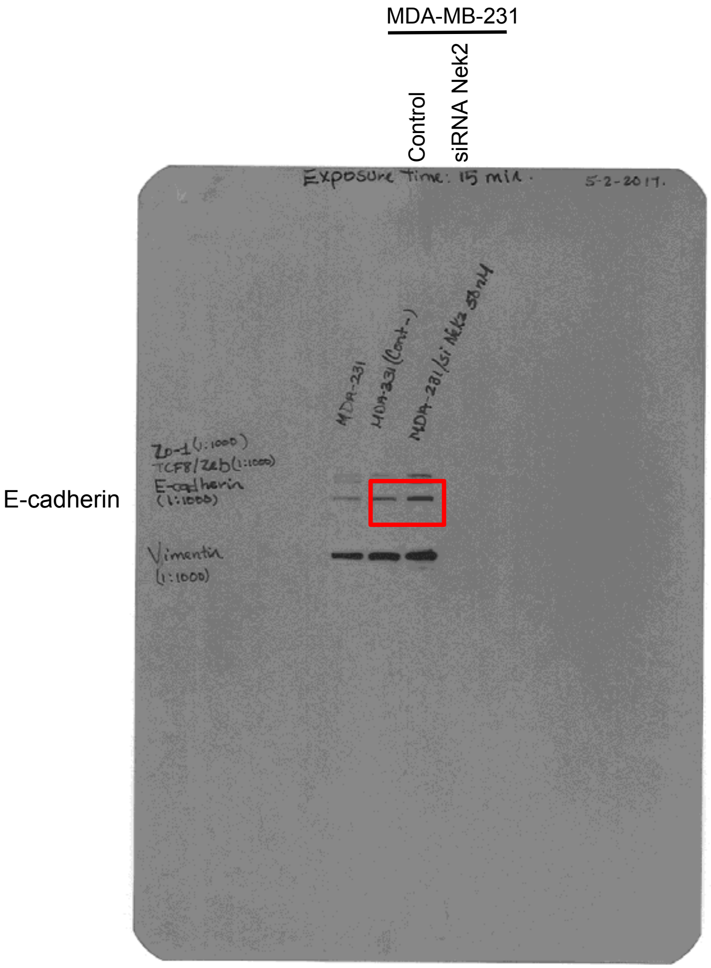

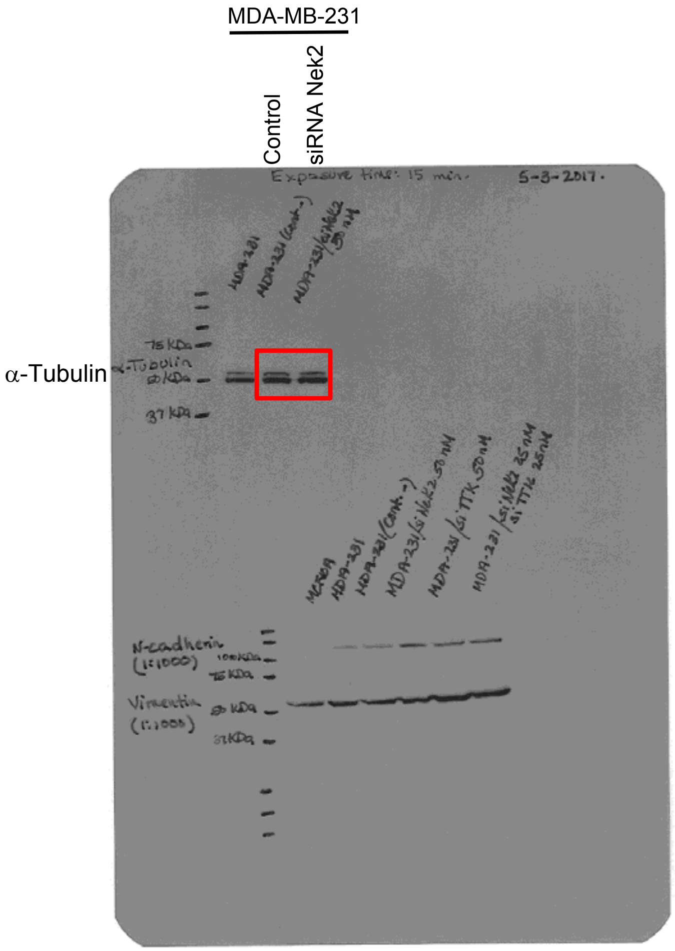

Original Western Blots for MDA-MB-231 and Hs578t (Fig. 4 a-b)

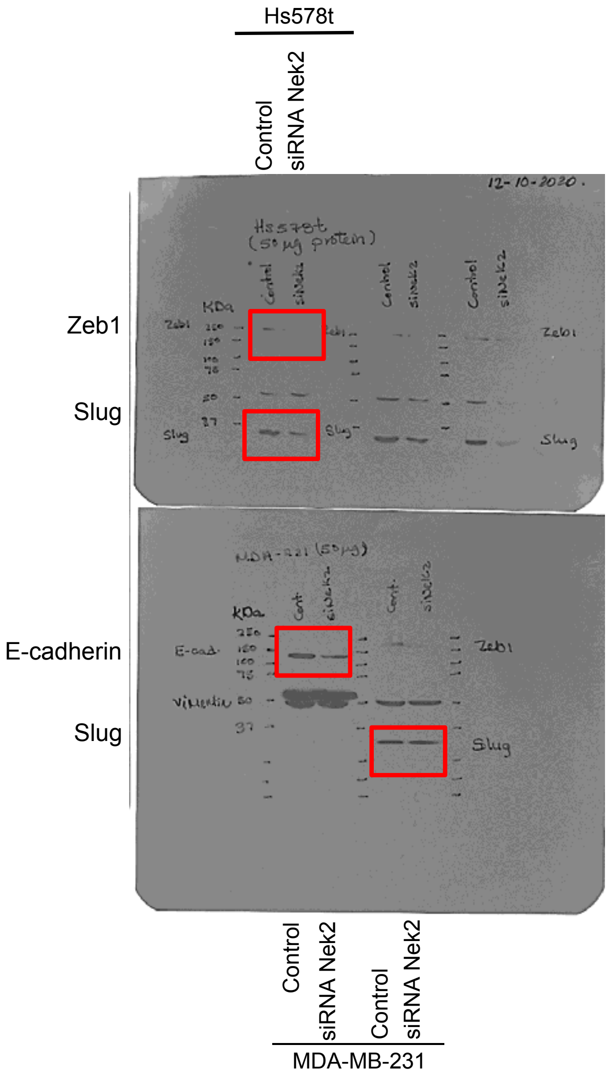

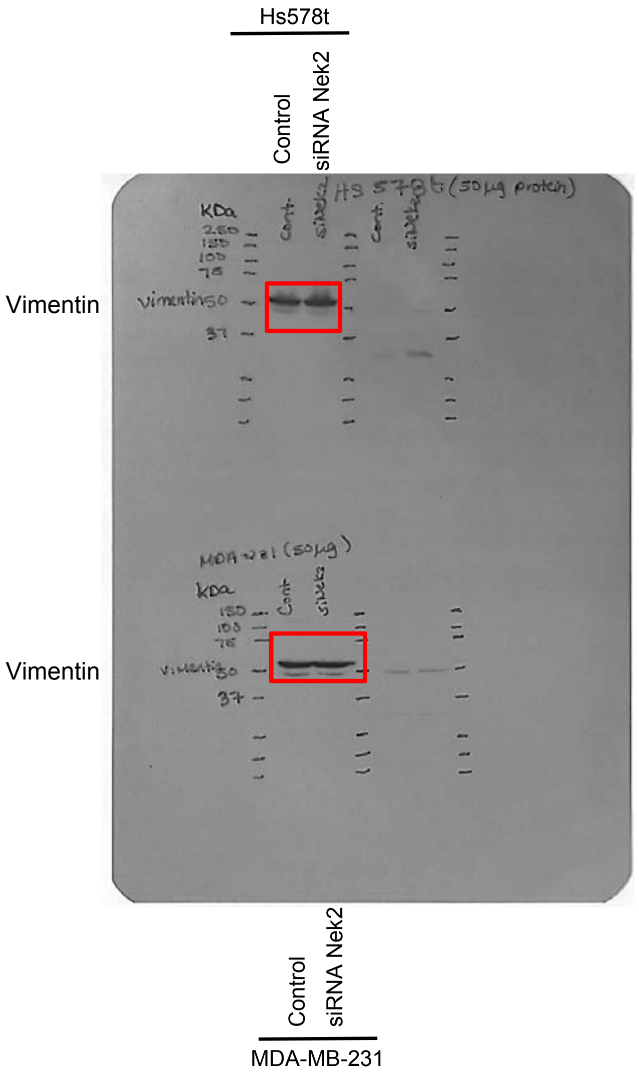

Original Western Blots for Fig. 4 a-b

Hs578t

N-Cadherin

β-actin

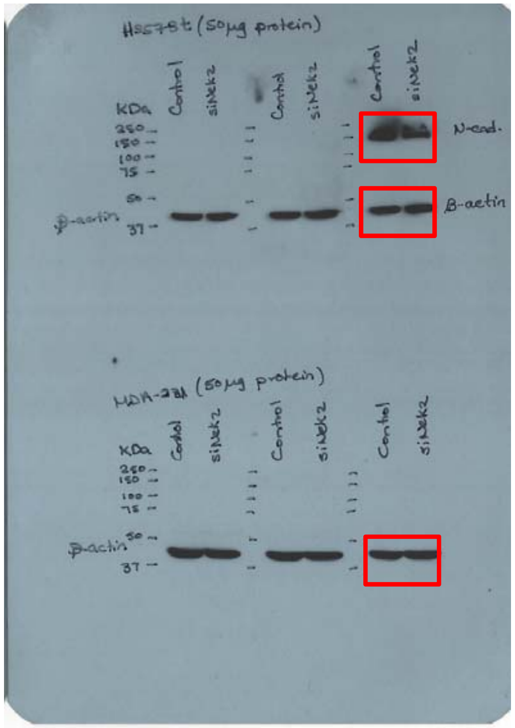

β-actin

MDA-MB-231

Hs578t

Nek2

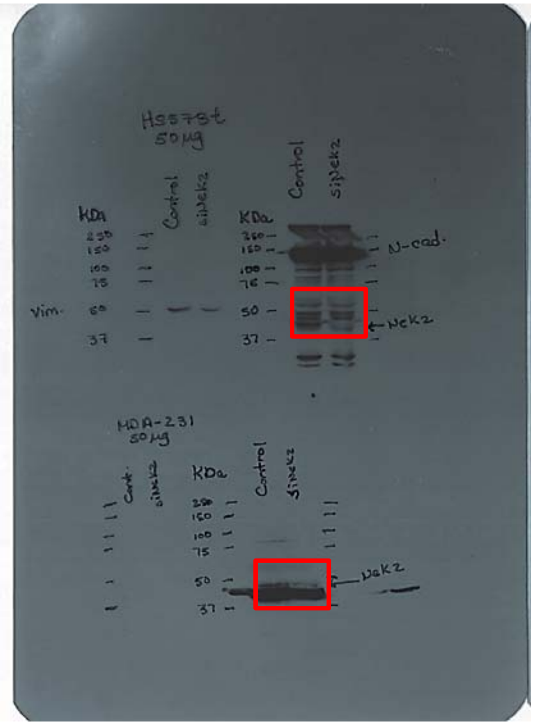

Nek2

MDA-MB-231

Original Western Blots used for Supplementary Figure 6

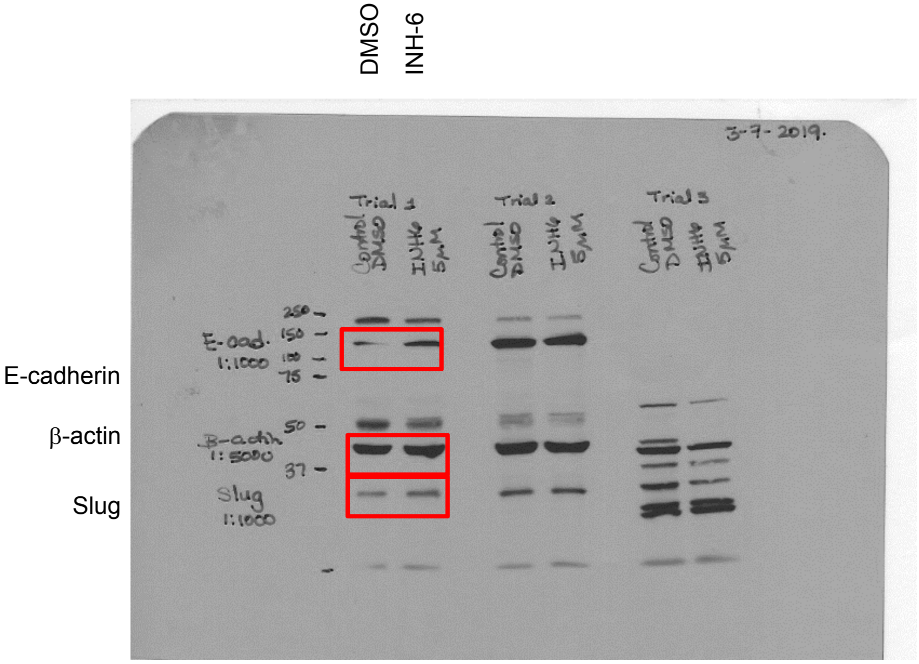

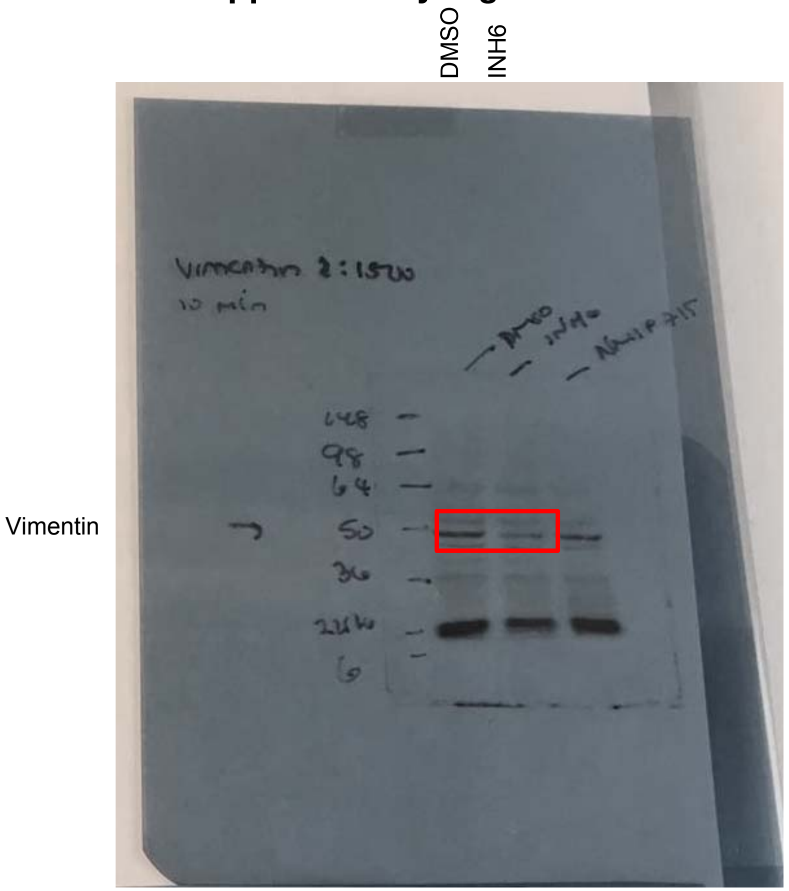

Original Western Blots used for Supplementary Figure 6

DMSO
INH6

α-tubulin
β-actin

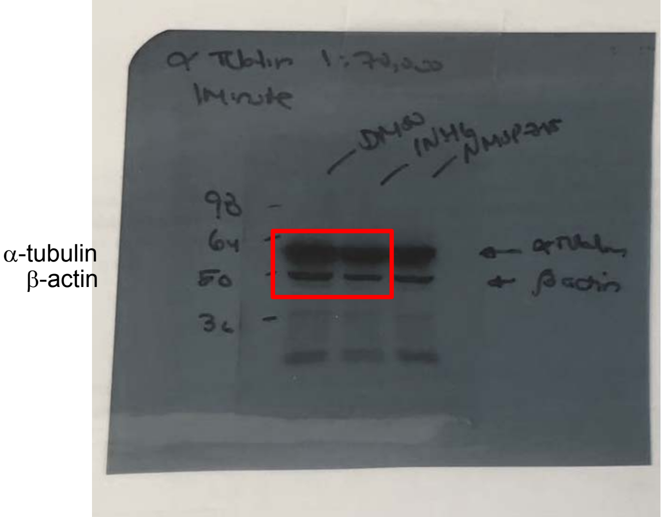

Supplement: Supplementary file 1 — Supplementary Information. [file 41598_2021_88512_MOESM1_ESM.pdf]
